# Three-Dimensional Structure Characterization and Inhibition Study of Exfoliative Toxin D From *Staphylococcus aureus*

**DOI:** 10.3389/fphar.2022.800970

**Published:** 2022-02-18

**Authors:** Anwar Ullah, Ajmal Khan, Ahmed Al-Harrasi, Kifayat Ullah, Asghar Shabbir

**Affiliations:** ^1^ Department of Biosciences COMSATS University Islamabad, Islamabad, Pakistan; ^2^ Natural and Medical Sciences Research Center, University of Nizwa, Nizwa, Oman

**Keywords:** *Staphylococcus aureus*, exfoliative toxins, exfoliative toxin D, comparative protein modeling, three-dimensional structure characterization, inhibition

## Abstract

The *Staphylococcus aureus* exfoliative toxins (ETs) are the main toxins that produce staphylococcal scalded skin syndrome (SSSS), an abscess skin disorder. The victims of the disease are usually newborns and kids, as well as grown-up people. Five ETs namely, exfoliative toxins A, B, C, D, and E have been identified in *S. aureus*. The three-dimensional (3D) structure of exfoliative toxins A, B, C and E is known, while that of exfoliative toxin D (ETD) is still unknown. In this work, we have predicted the 3D structure of ETD using protein modeling techniques (software used for 3D structure modeling comprising the MODELLER 9v19 program, SWISS-Model, and I-TESSER). The validation of the build model was done using PROCHECK (Ramachandran plot), ERRAT2, and Verify 3D programs. The results from 3D modeling show that the build model was of good quality as indicated by a GMQE score of 0.88 and by 91.1% amino acid residues in the most favored region of the Ramachandran plot, the ERRAT2 quality factor of 90.1%, and a verify3D score of >0.2 for 99.59% of amino acid residues. The 3D structure analysis indicates that the overall structure of ETD is similar to the chymotrypsin-like serine protease fold. The structure is composed of 13 β-strands and seven α-helices that fold into two well-defined six-strand β-barrels whose axes are roughly perpendicular to each other. The active site residues include histidine-97, aspartic acid-147, and serine-221. This represents the first structure report of ETD. Structural comparison with the other ETs shows some differences, particularly in the loop region, which also change the overall surface charge of these toxins. This may convey variable substrate specificity to these toxins. The inhibition of these toxins by natural (2S albumin and flocculating proteins from *Moringa oleifera* seeds) and synthetic inhibitors (suramin) was also carried out in this study. The results from docking indicate that the inhibitors bind near the C-terminal domain which may restrict the movement of this domain and may halt the access of the substrate to the active site of this enzyme. Molecular dynamic simulation was performed to see the effect of inhibitor binding to the enzyme. This work will further elucidate the structure–function relationship of this enzyme. The inhibition of this enzyme will lead to a new treatment for SSSS.

## Introduction

Exfoliative toxins (ETs) of *Staphylococcus aureus* are the main causative agents of staphylococcal scalded skin syndrome (SSSS) (Hubiche et al., 2012; Kimberlin et al., 2015; Nguyen et al., 2021). The syndrome is characterized by a red rash and dissolution of the skin lying below the granular cell layer (Ladhani, 2003; Hanakawa and Stanley, 2004; Sahin-Tóth et al., 2021). The sites of infection include oral or nasal cavities, and the throat and umbilicus (Ladhani, 2003; Adhisivam and Mahadevan, 2006; Abril et al., 2020). The clinical symptoms start suddenly with fever, skin soreness, and itches, within a few hours to a few days of the infection (Amagai et al., 2000; Del Giudice, 2020). There are two common forms (localized and generalized) of SSSS that have been reported in the literature. The localized form (also called bullous impetigo) is recognized by the formation of minor patches on the skin, while the generalized form is characterized by the involvement of a large surface from the skin (Lyell, 1979; Cribier et al., 1984; Ladhani et al., 1999; Mannschreck et al., 2020; Del Giudice, 2020). Children under the age of 5 years are more susceptible to SSSS as compared to adults, due to the lack of removal for the exotoxins from the circulating system and the immature immune system (Oono et al., 1997; Shirin et al., 1998; Kapoor et al., 2008; Kadam et al., 2009; Doudoulakakis et al., 2021). Both of these are more developed in adults. The adults also have antibodies that specifically neutralize the toxins; however, in children, no such antibodies exist (Decleire et al., 2004). The SSSS-infected adults may show some symptoms like kidney dysfunction, immunodeficiency, and other long-term illness (Ladhani and Newson 2000; Decleire et al., 2004; Nguyen et al., 2021; Schwarz et al., 2021). The lethality rate from SSSS is under 5% (Melish, 1982; Cribier et al., 1984).

The ETs are highly specific toward their substrates, and these only cleave desmoglein 1 (Amagai et al., 2000; Amagai et al., 2002; Yamaguchi et al., 2002) and the cleavage occurs in a calcium-dependent confirmation of desmoglein 1 (Hanakawa et al., 2003). The removal of calcium from desmoglein 1, or pretreatment at 56°C or higher, or at low or high pH changes the conformation of desmoglein 1 and the ETs become unable to cleave it (Hanakawa et al., 2003). There is one unique site (following the glutamic acid at amino acid position 381) that is specifically cleaved by ETs in Dsg1 in mice and humans, and the ETs are unable to cleave identical substrates like Dsg3 and E-cadherin (Amagai et al., 2000; Hanakawa et al., 2002).

The ETs are serine proteinases with exotoxins enzymatic activity (Ladhani, 2003). Five types of ETs have been described in the literature, namely, exfoliative toxin A (ETA) (Cavarelli et al., 1997; Azarian et al., 2021), exfoliative toxin B (ETB) (Gemmell, 1997), exfoliative toxin C (ETC) (Sato et al., 1994), exfoliative toxin D (ETD) (Yamaguchi et al., 2002), and exfoliative toxin E (ETE) (Imanishi et al., 2019). The primary amino acid sequences of these contain 242–247 amino acid residues in their mature form (Cavarelli et al., 1997; Papageorgiou et al., 2000; Ladhani, 2003; Mariutti et al., 2015). The sequence identity among ETA, ETB, ETD, and ETE ranges from 43 to 63% (Ladhani, 2003); however, with ETC, these do not display any significant sequence identity (Ladhani, 2003). ETC, also known as adenylosuccinate lyase, is not toxic to humans, and its three-dimensional (3D) structure is also different from ETA, ETB, and ETE (Fyfe et al., 2010).

The crystal structures of ETA, ETB, ETC, and ETE have been described (Cavarelli et al., 1997; Papageorgiou et al., 2000; Fyfe et al., 2010; Mariutti et al., 2015). The crystal structure of ETD-like protein (Mariutti et al., 2015) described previously has now been renamed as ETE (Imanishi et al., 2019). The ETD-like protein has 59% sequence identity to ETD, and that is why it is called ETD-like (Imanishi et al., 2019). The 3D structure of ETs is similar to the classical serine proteinase, chymotrypsin-like fold (Cavarelli et al., 1997; Papageorgiou et al., 2000; Mariutti et al., 2015). Their 3D structure is composed of 13 β-strands and seven α-helices that fold into two well-organized six-stranded β-barrels whose axes are roughly perpendicular to each other (Cavarelli et al., 1997; Papageorgiou et al., 2000; Mariutti et al., 2015). The active site is positioned at the intersection of the two β-barrels, comprising of amino acid residues His-Asp-Ser, which are fully conserved in all the ETs and with other serine proteinases (Cavarelli et al., 1997; Papageorgiou et al., 2000; Mariutti et al., 2015).

Although the crystal structures of the ETs A, B, C, and E are known, the crystal structure of ETD is still unknown. This lack in the structure of ETD makes it difficult to establish a structure–function relationship and the mechanism of action and inhibition of this enzyme. The literature review indicates that most of the research work done on the ETD belongs to gene-level identification and characterization and the mode of spreading (Yamasaki et al., 2006; Mališová et al., 2020; Doudoulakakis et al., 2021). To better understand their 3D structure and mechanism of action, the present work depicts the model-based structure elucidation and characterization of ETD from *S. aureus*. A structure-based mechanism of action and inhibition study was carried out for ETD. Although this study is *in silico*, it will provide a better way to perform it in the lab for further verification.

## Materials and Methods

### Sequence Extraction and Multiple Sequence Alignment

The primary amino acid sequence of ETD was retrieved from the National Centre for Biotechnology Information (NCBI) protein database (http://www.ncbi.nlm.nih.gov/protein), with the Gene Bank ID: AHC54578.1 (1–281 amino acid residues) (Paul et al., 2014) and the corresponding UniProt ID: Q8GAX8.

### Prediction of Signal Peptide

The signal peptide in the primary structure of ETD was predicted using the program SignalP 3.0 server (Bendtsen et al., 2004), by providing the full-length amino acid sequence of ETD. The parameters used were organism group—Gram-positive bacteria and method—both neural network and Hidden Markov models, and the sequence was truncated to a maximum of 50 amino acid residues.

### Biochemical Properties of the Exfoliative Toxin D

The molecular weight and isoelectric point (p*I*) of ETD was computed using the program ProtParam tools from the ExPASy Proteomics server (http://web.expasy.org/compute_pi/) (Gasteiger et al., 2005).

### Generation of Sequence Logo From Multiple Sequence Alignment

For the creation of the sequence logo from the aligned sequences, WebLogo 3.2 (Crooks et al., 2004; Schneider and Stephens, 1990) was used by providing the default parameters.

### Prediction of Glycosylation Sites

The potential glycosylation sites were predicted using the online webserver NetNGlyc 1.0 (Gupta et al., 2004), with parameters set to default.

### Homology Model Building

For the generation of 3D homology model, we used numerous web-based protein modeling programs, including I-TESSER, MODELLER 9v19 program, and SWISS MODEL (Roy et al., 2010; Webb and Sali, 2016; Waterhouse et al., 2018). The atomic coordinates of ETE, with 63.71% identity (PDB ID: 5C2Z; Mariutti et al., 2015) was employed as a template.

### Model Quality Validation and Assessment

The build ETD 3D model was validated and assessed by employing programs like PROCHECK (Laskowski et al., 2001; Laskowski et al., 1993), ERRAT version 2.0 (Colovos and Yeates, 1993), and Verify 3D (Bowie et al., 1991; Lüthy et al., 1992).

### Molecular Docking

The protein and ligands were prepared for molecular docking by removing water molecules from the protein and adding hydrogen atoms. The ionization states of the atoms were kept in the ligand as mentioned in the database. The ligand geometry was optimized using the AM1 method (Dewar et al., 1985). The AM1-BCC method was used for calculating the partial charges of the ligands (Jakalian et al., 2002). The general AMBER force field (GAFF) method (Wang et al., 2004) was used for assigning the atoms type, bond angle, dihedral, and van der Waals parameters for the ligands. For molecular docking of the protein and ligands, the programs used were Molecular AutoDock 4.0, HADDOCK2.4, patchdock, and pardock (Schneidman-Duhovny et al., 2005; Gupta et al., 2007; Morris et al., 2009; van Zundert et al., 2016). The refinement of the protein–ligand complex was done using the FireDock (Mashiach et al., 2008).

### Molecular Dynamics Simulation

The build 3D structure of ETD was assessed through various molecular dynamics (MD) simulation programs, like AMBER16 (Maier et al., 2015), GROMACS (Berendsen et al., 1995), MDweb, and MDMoby (Hospital et al., 2012). The FF14SB force field was used to measure all-atom protein interaction (Darden et al., 1993). The web server H++ (Anandakrishnan et al., 2012) was used to calculate the protonation states of the amino acid side chain, at neutral pH (7.0). The chloride ions (Cl^−^) were used to neutralize the system. Then it was placed in a rectangular box of TIP3P water and expanded to at least 15 Å from any protein atom. The protein structure was optimized by minimizing the system for 500 conjugate gradient steps by applying a constant force constraint of 15 kcal/mol. The system was steadily heated from 0 to 300 K for 250 ps with a constant atom number, volume, and temperature all at once; simultaneously, the protein was restrained with a constant force of 10 kcal/mol Å2. The equilibration step was done using the constant atom number, pressure, and temperature ensemble for 500 ps, and the simulation was done for 100 ns with a 4 fs time step. The temperature and pressure were kept constant at 300 K and 1 atm, respectively, by Langevin coupling. The particle–mesh Ewald method (Darden et al., 1993) was used to compute the long-range electrostatic interactions. A cut-off distance of 10 Å to Van der Waals interactions was used during this process. The results from MD simulation were visualized using Visual Molecular Dynamics (Humphrey et al., 1996) and PyMOL (De Lano, 2002) molecular graphic visualization software.

### Structure Alignment

The ETD structure was aligned to the other identical proteins using the PyMOL molecular graphics visualization program (De Lano, 2002).

### Surface Charge Analysis

The PDB2PQR online server program was used to calculate the charge and radius (Dolinsky et al., 2004). The visualization of the surface and charge were carried out using the APBS tool from the PyMOL molecular graphics visualization program (De Lano, 2002).

## Results and Discussion

### Multiple Sequence Alignment Analysis

**FIGURE 1:**
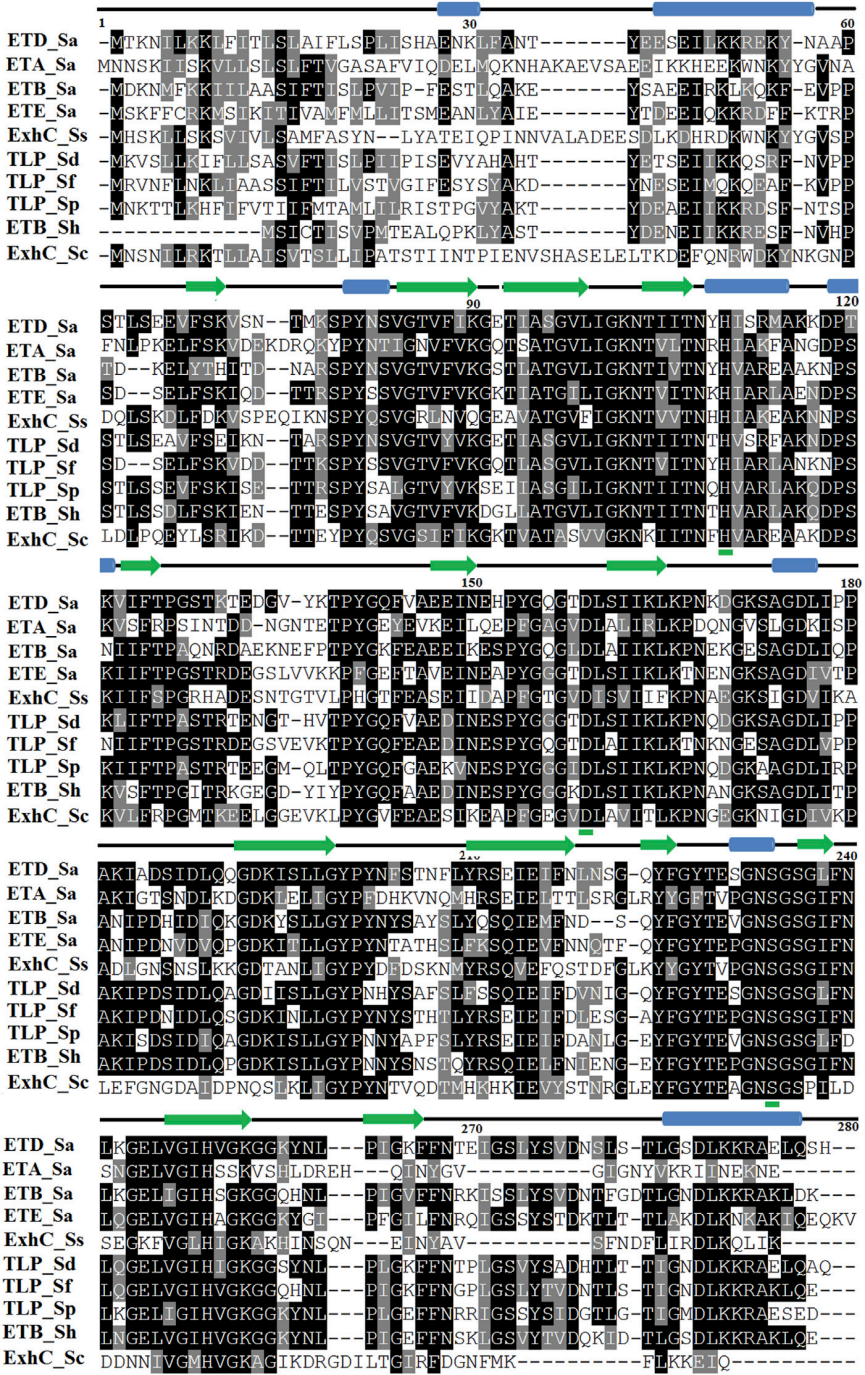
Sequence alignment of ETs. ETD_*Sa*: exfoliative toxin D, *Staphylococcus aureus*, Gene Bank ID: AHC54578.1; ETA_*Sa*: epidermolytic toxin A from *S. aureus*, PDB ID: 1AJGJ; ETB_*Sa*: crystal structure of exfoliative toxin B, PDB ID: 1DT2; ETE_*Sa*: exfoliative toxin E from *S. aureus*, Gene Bank ID: WP_054190843.1, PDB ID: 5C2Z; ExhC_Ss: exfoliative toxin ExhC from *S. sciuri*, Gene Bank ID AEF13380.1; TLP_*Sd*: trypsin-like peptidase domain–containing protein from *S. delphini*, Gene Bank ID: WP_096546202.1; TLP_*Sf*: trypsin-like peptidase domain–containing protein from *S. felis*, Gene Bank ID: WP_103209705.1; TLP_Sp: trypsin-like peptidase domain–containing protein *S. pseudintermedius*, Gene Bank ID: WP_100002848.1; ETB_*Sh*: exfoliative toxin B from *S. hyicus*, Gene Bank ID: BAA99411.1, ExhB_*Sc*: exfoliative toxin ExhB *S. chromogenes*, Gene Bank ID: AAV98626.1. Amino acid residues involved in catalysis underlined with green. Secondary structural elements (alpha helices and beta strands) are shown above the sequence. Sequence numbering corresponds to the ETD_Sa precursor protein.

The primary structure of ETD consists of 281 and 255 amino acid residues in the inactive (proenzyme) and active forms, respectively. The analysis of SignalP 3.0 shows that the first 26 amino acid residues of the proenzyme belong to the signal peptide (Supplementary Figure S1). The multiple sequence alignment analysis indicate a high sequence identity between ETD and the other ETs of *S. aureus* [ETA (50%), ETB (62.62%), and ETE (57.09%)] and ETs of *Staphylococcus sciuri* (ExhC, 45.16%), *Staphylococcus delphini* trypsin-like peptidase domain–containing protein (69.40%), *Staphylococcus felis* trypsin-like peptidase domain–containing protein (67.50%), *Staphylococcus pseudintermedius* trypsin-like peptidase domain–containing protein (65.36%), *Staphylococcus hyicus* ETB (66.60%), and *Staphylococcus chromogenes* ET ExhB (43.48%) (Table 1). The average sequence identity between ETD and other aligned ETs are 58.57%. The ETD shows very low sequence identity to *Staphylococcus epidermidis* glutamyl endopeptidases (30.59%). The amino acid residues of the catalytic triad (His110, Asp159, and Ser234) are completely conserved among all the aligned ETs (Figure 1). Besides the amino acid residues of the catalytic triad, the other fully conserved amino acid residues between ETD and others ETs include Pro80, Tyr81, Gly85, Gly100, Lys101, Asn102, Thr107, Asn108, Ala115, Pro119, Phe124, Pro126, Pro140, Gly 142, Pro153, Gly155, Gly157, Ileu163, Gly171, Gly175, Asp176, Ala181, Gly192, Asp193, Leu197, Gly199, Tyr200, Pro201, Glu215, Tyr225, Gly227, Thr229, Gly232, Asn233, Ser234, Gly235, Ser236, Gly247, and His249. The analysis of the sequence logo file produced using the WebLogo 3.2 indicates that the amino acids residues surrounding the active site are highly conserved among all the aligned ETs (Supplementary Figure S2).

**TABLE 1:** Percent sequence identities among ETD and other ETs.

Proteins	ETD_*Sa*	ETA_*Sa*	ETB_*Sa*	ETE_*Sa*	ExhC_*Ss*	TLP_*Sd*	TLP_*Sf*	TLP_*Sp*	ETB_*Sh*	ExhC_*Sc*
ETD_*Sa*	—	50%	62.62%	57.09%	45.16%	69.40%	67.50%	65.36%	66.60%	43.48%
ETA_*Sa*	50%	—	44.00%	43.35%	44.85%	42.98%	47.41%	43.32%	47.14%	38.61%
ETB_*Sa*	62.62%	44.00%	—	55.36%	37.19%	60.63%	64.64%	55.91%	59.48%	39.44%
ETE_*Sa*	57.09%	43.35%	55.36%	—	40.16%	56.38%	63.27%	58.91%	59.63%	43.03%
ExhC_*Ss*	45.16%	44.85%	37.19%	40.16%	—	40.41	46.88%	39.63%	44.24%	38.57%
TLP_*Sd*	69.40%	42.98%	60.63%	56.38%	40.41%	—	64.29%	64.29%	66.92%	39.29%
TLP_*Sf*	67.50%	47.41%	64.64%	63.27%	46.88%	64.29%	—	60.07%	67.16%	41.04%
TLP_*Sp*	65.36%	43.32%	55.91%	58.91%	39.63%	64.29%	60.07%	—	70.36%	39.13%
ETB_*Sh*	66.60%	47.14%	59.48%	59.63%	44.24%	66.92%	67.16%	70.36%	—	41.42%
ExhC_*Sc*	43.48%	38.61%	39.44%	43.03%	38.57%	39.29%	41.04%	39.13%	41.42%	—

ETD_*Sa*, exfoliative toxin D, *Staphylococcus aureus*, Gene Bank ID: AHC54578.1; ETA_*Sa*, epidermolytic toxin A from *S. aureus*, PDB ID: 1AJGJ; ETB_*Sa*, crystal structure of exfoliative toxin B, PDB ID: 1DT2; ETE_*Sa*, exfoliative toxin E from *S. aureus*, Gene Bank ID: WP_054190843.1, PDB ID: 5C2Z; ExhC_Ss, exfoliative toxin ExhC from *S. sciuri*, Gene Bank ID: AEF13380.1; TLP_*Sd*, trypsin-like peptidase domain–containing protein from *S. delphini*, Gene Bank ID: WP_096546202.1; TLP_*Sf*, trypsin-like peptidase domain–containing protein from *S. felis*, Gene Bank ID: WP_103209705.1; TLP_Sp, trypsin-like peptidase domain–containing protein from *S. pseudintermedius*, Gene Bank ID: WP_100002848.1; ETB_*Sh*, exfoliative toxin B from *S. hyicus*, Gene Bank ID: BAA99411.1; ExhB_*Sc*: exfoliative toxin ExhB from *S. chromogenes*, Gene Bank ID: AAV98626.1.

### Domain Analysis

The domain analysis carried out using conserved domain search (CD Search; Marchler-Bauer et al., 2017) and CDART (Conserved Domain Architecture Retrieval Tools; Geer et al., 2002) on the NCBI website indicate that the ETD is a single domain protein belonging to trypsin-like serine proteinases (Tryp_SPc super family; 61–236 amino acid residues matching) most of which are synthesized as inactive precursors or zymogens. These are converted to the active form after limited proteolysis.

The MotifFinder analysis indicates that ETD is a trypsin-2 peptidase and belongs to the trypsin-like peptidase domain (84–235 amino acid residues matching) (Supplementary Figure S3).

### Glycosylation

Although Rogolsky et al. (1974) have shown that ETs of *S. aureus* contain 9% carbohydrates; the NetGlycan 4.0 server (Steentoft et al., 2013) indicates that ETD is not glycosylated. The crystal structures of other ETs (A, B, and E) also do not have any glycosylation (Cavarelli et al., 1997; Papageorgiou et al., 2000; Mariutti et al., 2015).

### Biochemical Properties

The molecular weights of precursor and mature ETDs were 30.80 and 27.90 kDa, with corresponding p*I*s 8.90 and 7.99, respectively. These values are in agreement with the previously calculated values for ETs (De Azavedo et al., 1988; Gasteiger et al., 2005).

The Scoop (Pucci et al., 2017) analysis for protein thermal stability shows the melting temperature (Tm) of ETD is 56.5°C (Supplementary Figure S4), which is in agreement with the previous experimentally measured value for ETA, ETB, and ETE (Piemont et al., 1986; Mariutti et al., 2015).

### Homology Modeling, Model Evaluation, and Molecular Docking

For 3D structural characterization, the homology model of ETD was generated by applying various software including the SWISS MODEL (Waterhouse et al., 2018), MODELLER 9v19 program (Webb and Sali, 2016), and I-TESSER (Roy et al., 2010). The atomic coordinates of ETE, with 63.71% identity (PDB ID: 5C2Z; Mariutti et al., 2015) was employed as a template (Supplementary Figure S5). After a comparison of the build models from all the three programs, the model built by the SWISS MODEL was selected based on GMQE which depicts the quality estimation of it (Supplementary Tables S1, S2). The GMQE score is given as a number between 0 and 1. Higher numbers show higher accuracy. So, the model has the GMQE value of 0.88, reflecting good accuracy of the model. The build model of ETD was validated using PROCHECK (Ramachandran plot) (Laskowski et al., 1993), ERRAT2 (Colovos and Yeates, 1993), and Verify3D (Bowie et al., 1991; Lüthy et al., 1992). The Ramachandran plot analysis shows that 91.1% of the amino acid residues were in the most favored region and 8.8% were in the additionally allowed region with no amino acid residue in generously allowed and disallowed regions (Supplementary Figure S6). The ERRAT2 inspection indicates the overall quality factor of 90.13%, which comes in the average range of protein 3D structure quality according to the program developer (Supplementary Figure S7). The results obtained from Verify3D display 99.59% of amino acid residues having a 3D–1D score >0.2 (Supplementary Figure S8). No poor area (zero or negative) in the ETD build model was detected by the Verify3D program, indicating a suitable environment for each amino acid residue in the determined structure (Supplementary Figure S8). The entire structural criterion including chirality and unusual *cis/trans* configuration was perfect, and there were no steric clashes in the build model (Supplementary Figure S9A), as indicated by results coming from molecular dynamic simulation (MDWeb analysis). The radius of gyration was constant at 68.8–68.9 Å during the whole process, with only 0.1 Å variations (Supplementary Figure S9B). The B-factor and RMSD per residue values indicate some regions with flexibility, mostly confined to the loop region of ETD structure (Supplementary Figures S9C,D).

The molecular docking results produced by AutoDock 4.0, HADDOCK2.4, patchdock, and pardock (Schneidman-Duhovny et al., 2005; Gupta et al., 2007; Morris et al., 2009; van Zundert et al., 2016) were nearly the same; however, we select the results from HADDOCK2.4 based on the RMSD value and various forms of energy released during the docking process (Supplementary Figures S13A,D, S14A). Also, the results from HADDOCK2.4 were very similar to MD simulation results from GROMACS, both of which give the same number of H-bonds between the ETD_*Sa* and the ligands (Supplementary Figures S13C,F; Figure 11).

### Overall Structure

**FIGURE 2:**
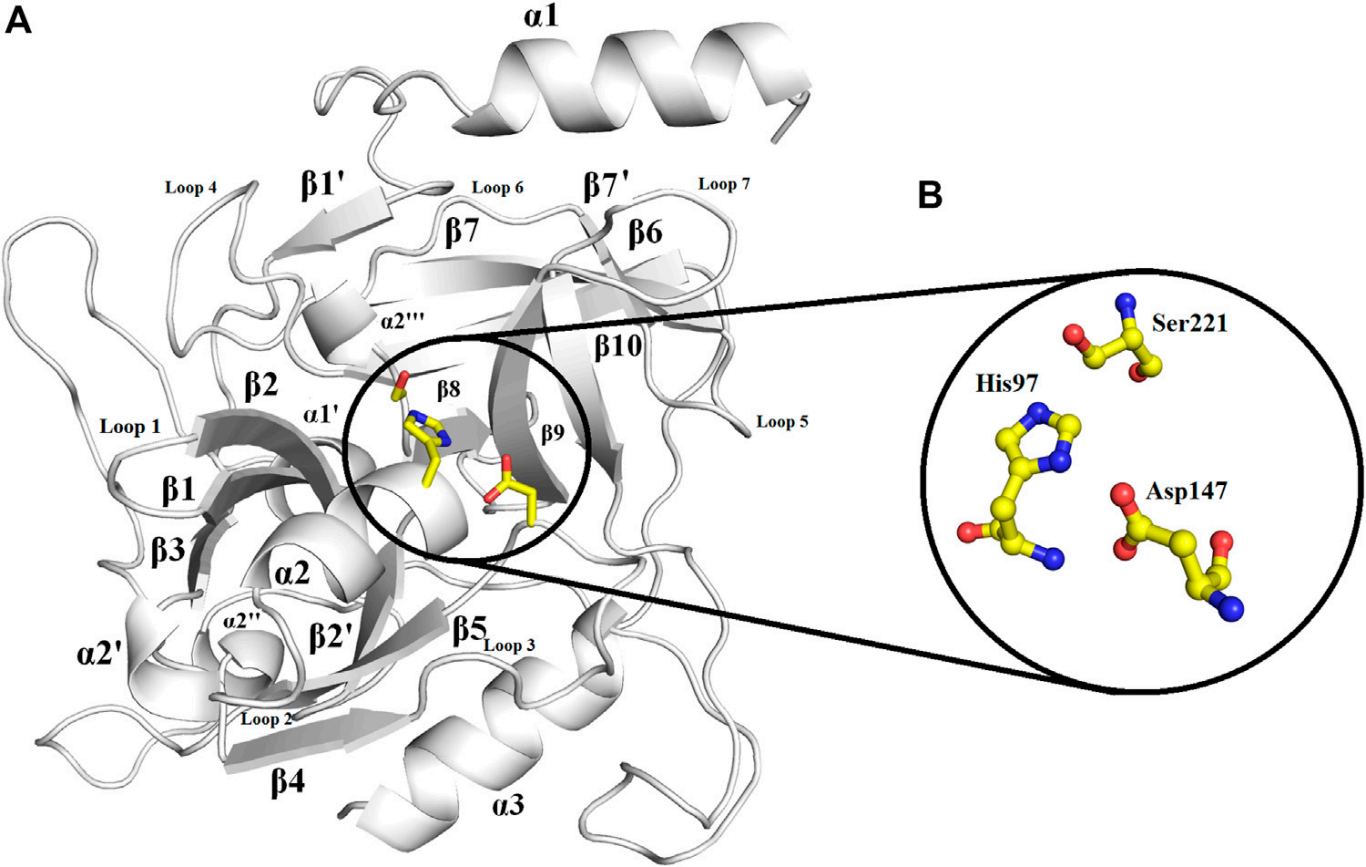
Overall structure of ETD_Sa: **(A)** Cartoon representation. The active site residues are depicted as yellow sticks. The loops surrounding the active site cavity are also indicated **(B).** Residues involved in catalysis (catalytic triad) are highlighted. Numbering is according to mature ETD_Sa.

The overall structure of ETD is similar to the chymotrypsin-like serine protease fold. The structure is composed of 13 β-strands and seven α-helices (Figure 2; Supplementary Figure S10) that fold into two well-defined six-stranded β-barrels whose axes are roughly perpendicular to each other. The β-strands are antiparallel to each other. To make its structure simpler for general discussion, it can be split into two domains, namely, N-terminal and C-terminal domains.

**FIGURE 3:**
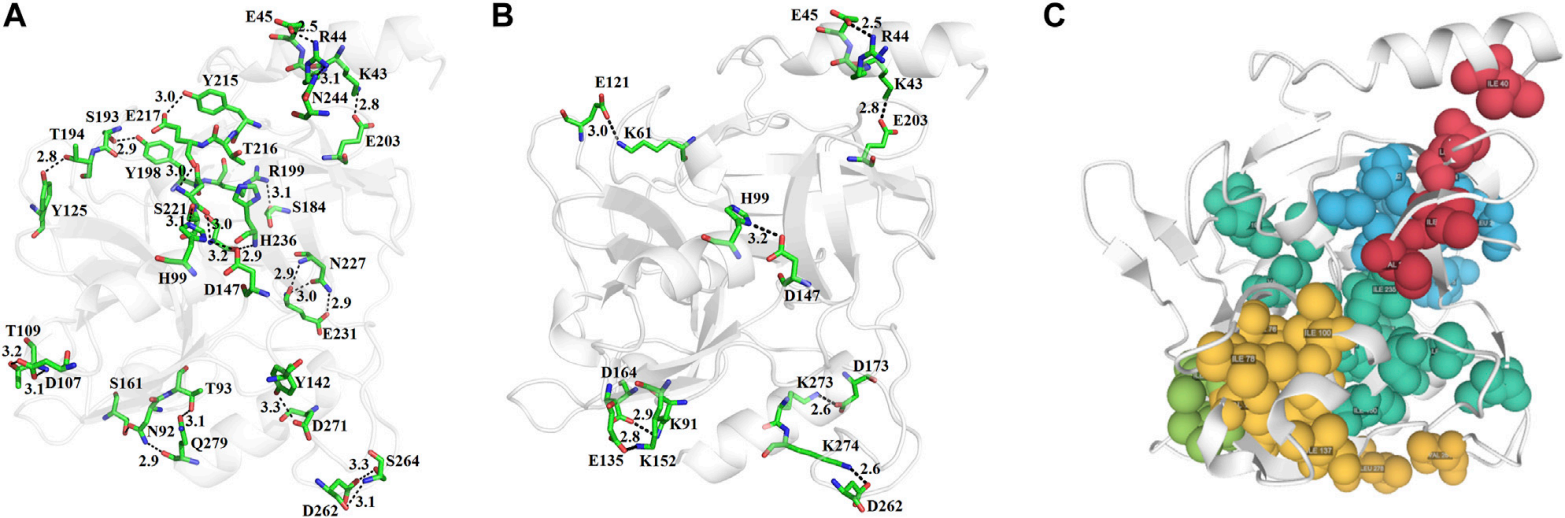
**(A)** Hydrogen bonds, **(B)** disulfide bridges, and **(C)** hydrophobic interactions in ETD_*Sa* 3D structure.

The N-terminal domain is composed of amino acid residues from Tyr35 to Leu148 and Leu228 to Lys242, from the C-terminal part of the protein. This domain comprises four α-helices (α1, α1′, α2, and α2′) and seven β-strands (β1′, β1, β2′, β2, β3, β4, and β9) (Supplementary Figure S10). This domain starts with a long α-helix which lies adjacent to the C-terminal β-barrel (Figure 2A). This alpha helix is charged having both acidic and basic amino acid residues (Asp, Glu, Lys, and Arg). The C-terminal domain is made up of amino acid residues from Ser149 to Asn227 and Tyr243 to Ser280. This domain contains three α-helices (α2″, α2‴, and α3) and five β-strands (β5, β6, β7, β7′, and β8) (Supplementary Figure S10). The active site which is composed of the three catalytic residues (catalytic triads) His, Asp, and Ser is located at the junction of the two domains (Figures 2A,B). The active site is surrounded by various loops from all four sides (Figure 2A). In several chymotrypsin-like serine proteinases, the loops 1, 2, 3, and 4 are known to participate in the determination of subsite preference, while loops 5, 6, and 7 are important to influence the specificity of the S1 site (Perona, and Craik, 1995). Although, like other serine proteinases, there are no disulfide bridges in the structure of ETD, the 3D structure of this enzyme (and other ETs) is highly stable due to various hydrogen bonds, salt bridges, and hydrophobic interactions (Figures 3A–C; Table 2).

**TABLE 2:** Hydrogen bonds, salt bridges, and hydrophobic interactions in the 3D structure of ETD_*Sa*.

H-bonds			
Network ID	Donor–acceptor	Distance	Angle DHA (degrees)
1	LYS43-NZ–GLU203-OE1	2.85	133.63
2	ARG44-NH2–GLU45-OE2	2.52	148.13
3	HIS99-ND1–ASP147-OD2	3.17	156.55
4	HIS99-NE2–SER221-OG	3.11	141.01
5	ARG199-NH2–SER184-OG	3.08	149.51
6	ASN220-ND2–SER223-OG	3.15	161.2
7	ASN227-ND2–GLU231-OE1	2.89	172.47
8	ASN244-ND2–ARG44-NH1	3.08	160.42
9	GLN279-NE2–THR93-OG1	2.92	137.44
10	THR109-OG1–ASP107-OD1	3.19	161.11
11	THR109-OG1–ASP107-OD2	3.32	142.7
12	TYR125-OH–THR194-OG1	2.8	167.92
13	TYR142-OH–ASP271-OD2	3.27	172.3
15	SER161-OG–ASN92-OD1	3.01	158.93
16	TYR198-OH–SER193-OG	2.9	176.31
17	TYR215-OH–GLU217-OE2	2.95	172.48
18	THR216-OG1–HIS236-NE2	3.2	152.06
19	SER264-OG–ASP262-OD2	3.14	161.6

Salt bridges		
Residue 1	Residue 2	Distance (Å)
NZ LYS A 43	OE1 GLU A 203	2.8
NH2 ARG A 44	OE1 GLU A 45	2.5
NZ LYS A 61	OE1 GLU A 121	3.0
NZ LYS A 91	OD2 ASP A 164	2.9
NE2 HIS A 99	OD2 ASP A 147	3.2
OE1 GLU A 135	NZ LYS A 152	2.8
OD2 ASP A 174	NZ LYS A 273	2.6

Hydrophobic interactions				
Cluster Id	Area (A)	No. contacts	Contacts/residue	Area (A)/residue
0	63.6	2	1.0	31.8
1	1922.9	49	3.3	39.2
2	926.2	21	2.6	44.1
3	62.9	2	1.0	31.5
4	75.1	2	1.0	37.6
5	1482.3	27	3.0	54.9
6	37.0	2	1.0	18.5
7	157.6	2	1.0	78.8

The PredictProtein server (Rost et al., 2004; Bigelow et al., 2004) indicates that the secondary structure of ETD is composed of 7.83% α-helices, 24.56% β-strands, and 67.62% loops (Supplementary Figure S11A). The results obtained from this program also showed that ETD has 43.06% amino acid where the residues are surface exposed, 51.63% are buried, and 5.34% are intermediate (Supplementary Figure S11B). These results are in agreement with the results obtained for other ETs (Mariutti et al., 2015).

### Active Site

**FIGURE 4:**
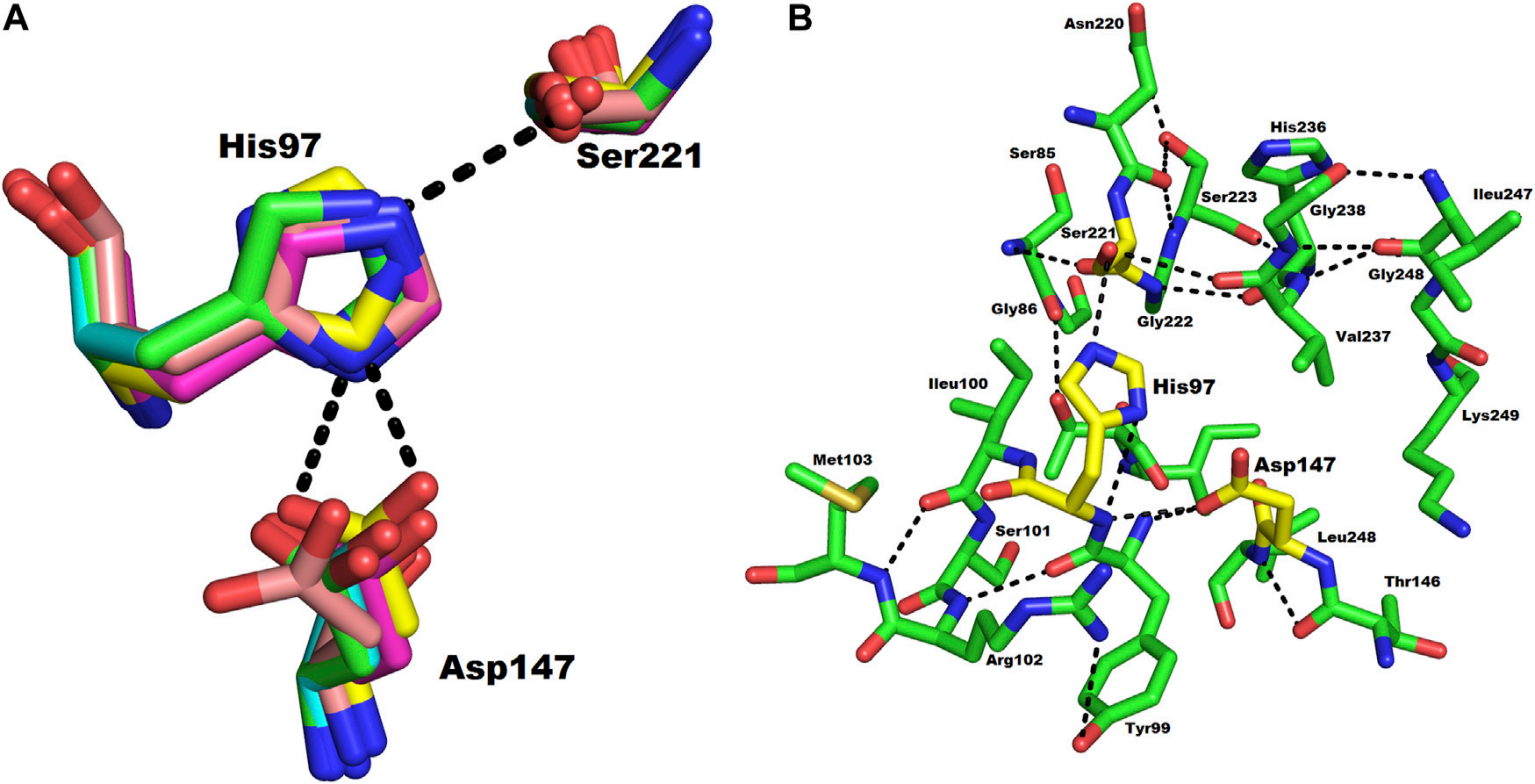
**(A)** Structural alignment of the three catalytic residues (catalytic triad) of ETs. Green, magenta, yellow, and cyan represents the triad of ETD_*Sa* (model), ETA, ETB, and ETE, respectively. **(B)** Hydrogen bond network in the catalytic triad of ETD_*Sa*. The amino acid residues are shown as green sticks.

The active site of ETD is made up of three amino acid residues (His, Asp, and Ser) which are called the catalytic triad. These three amino acid residues are fully conserved among all the aligned ETs (Figure 1). These amino acid residues are also aligned well structurally to each other (Figure 4A). The amino acid residues of the active site are stabilized by hydrogen bonds among themselves and with other amino acids residues nearby (Figure 4B). The His97 is H-bonded to Ser221, while Asp147 is bonded to the main chain of His97 and also to Tyr99, and Ser221 to the main chain of Val237 (Figure 4B).

### Metal Ion/Ligand Binding Sites

The analysis of the results forms Ion Com–Ion Ligand binding site prediction (Hu et al., 2016), identifies the amino acid residues (Glu5, Tyr64, His65, Glu101, His106, Asp147, Glu167, Phe171, Asn172, Phe179, Tyr181, Glu183, Ser184, G185, Asn186, Ser187, His202, Arg241, Ala242, Glu243, Leu244, Gln245, and Ser246 as potential binding sites for Zn^+2^, and Ileu49, Ala50, His65, Ileu66, Tyr181, Thr182, Glu183, Ser184, Gly185, Asn186, Ser187, His202, Val203, Gly204, Lys205, and Pro212 as potential binding sites for K^+^). Zn^+2^ and K^+^ have been also encountered in the 3D structure of an alkaline form of v8 proteinase from *S. aureus* (PDB ID: 1WCZ, unpublished work, PDB ID: 1QY6, Prasad et al., 2004). In this v8 proteinase, Zn^+2^ is tetrahedrally coordinated by Asp7, His9, and Lys147 (Supplementary Figure S12A), while K^+^ is coordinated by His107 only (Supplementary Figure S12B).

### Structural Comparisons Among Exfoliative Toxins

**FIGURE 5:**
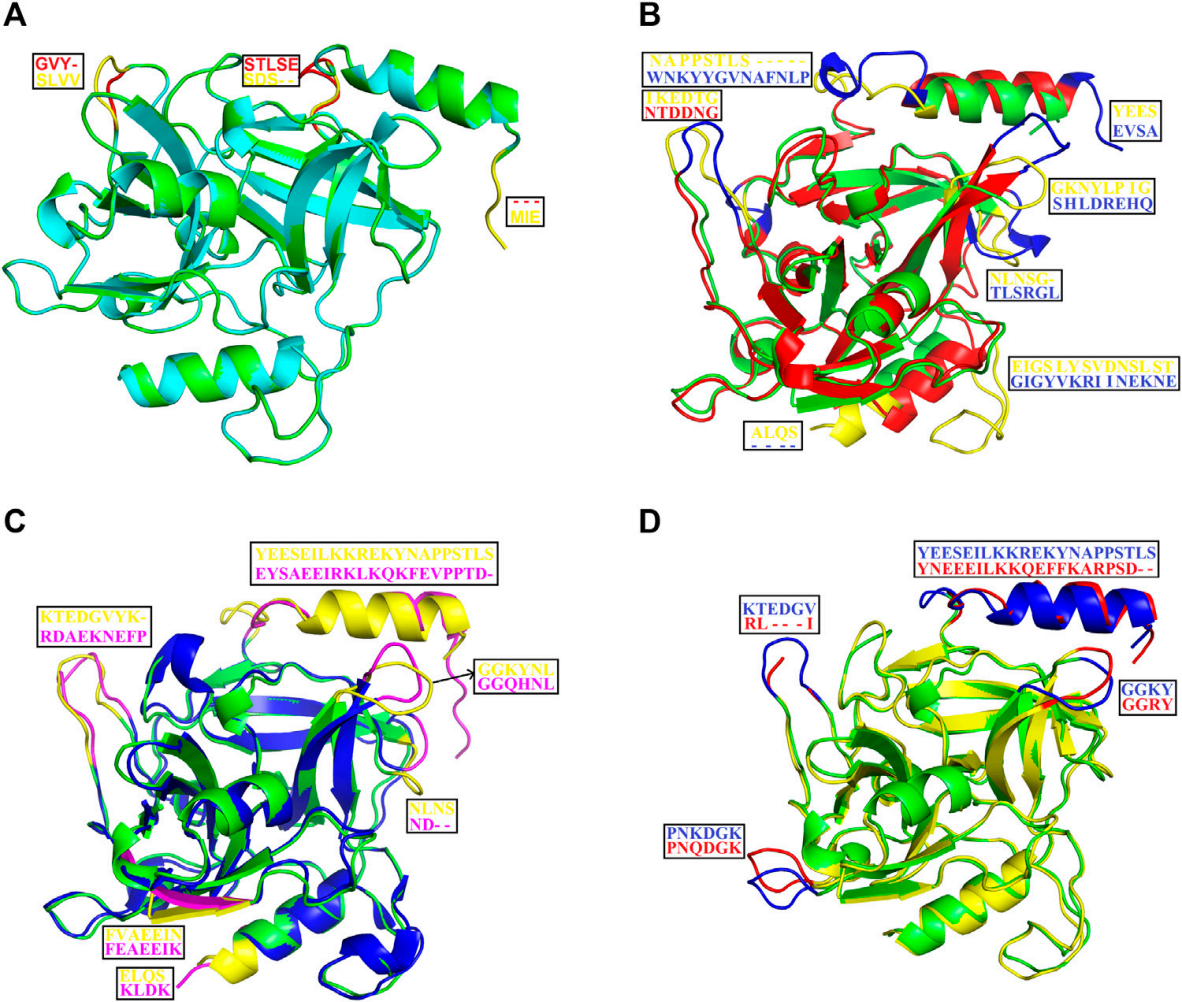
Structural alignment among ETS. **(A)** ETD_Sa (green) aligns with ETE_ Sa (cyan). **(B)** ETD_Sa (green) aligns with ETA_ Sa (red). **(C)** ETD_Sa (green) aligns with ETB_ Sa (blue). **(D)** ETD_Sa (green) align ET_ Sp (yellow) (*S. pseudintermedius* exfoliative toxin EXI). The regions (loops) displaying differences and their corresponding amino acid residues (shown in the box) are colored in yellow, pink, blue, and red.

**FIGURE 6:**
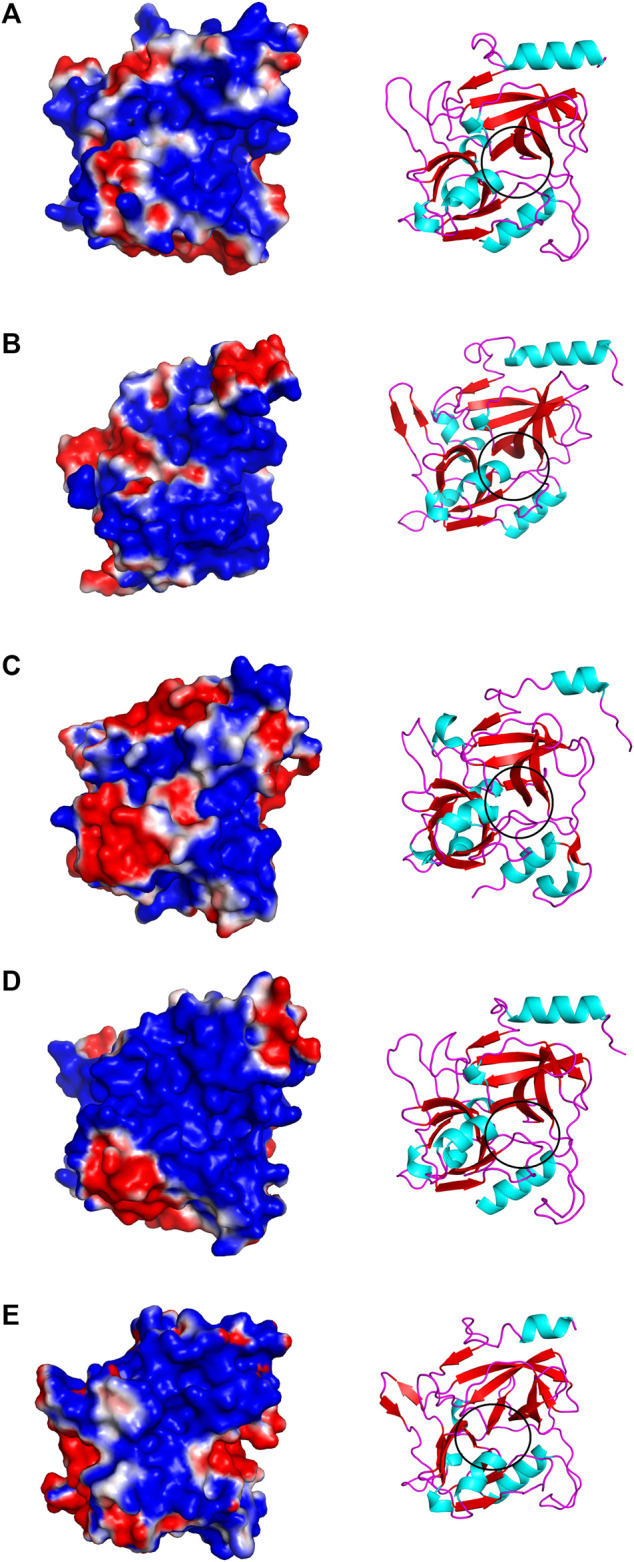
Surface charge distribution of **(A)** ETD_model, **(B)** ETA, **(C)** ETB, **(D)** ETE, and **(E)** ET_Exi; the active sites of each toxin is encircled.

The structural alignment analysis between ETD and other ETs indicates that all of these align well to each other with some differences in the loop regions (Figures 5A–D). The highest differences were found with ETA and ETB with an RMSD value of 0.618 and 0.632, respectively (Table 3). These differences were mostly due to amino acid residues confined to the loop regions (Figures 5A–D). The surface charge distribution of these enzymes also varies considerably because of these variations in the amino acid residues in the loop regions (Figures 6A–E). The surface charge of ETD is partially positive and partially neutral around the active site (Figure 6A) while that of ETA is partially positive and partially negative (Figure 6B). Similarly, the Figure 6C surface charge of ETB is highly negative (Figure 6C) and that of ETE is highly positive around the active site (Figure 6D).

**TABLE 3:** Root mean square deviation for structural alignment between ETD_*Sa* and other ETS.

Proteins	RMSD (Å)
ETD aligned ETE	0.060
ETD aligned ETA	0.618
ETD aligned ETB	0.632
ETD aligned EXI	0.516

**FIGURE 7:**
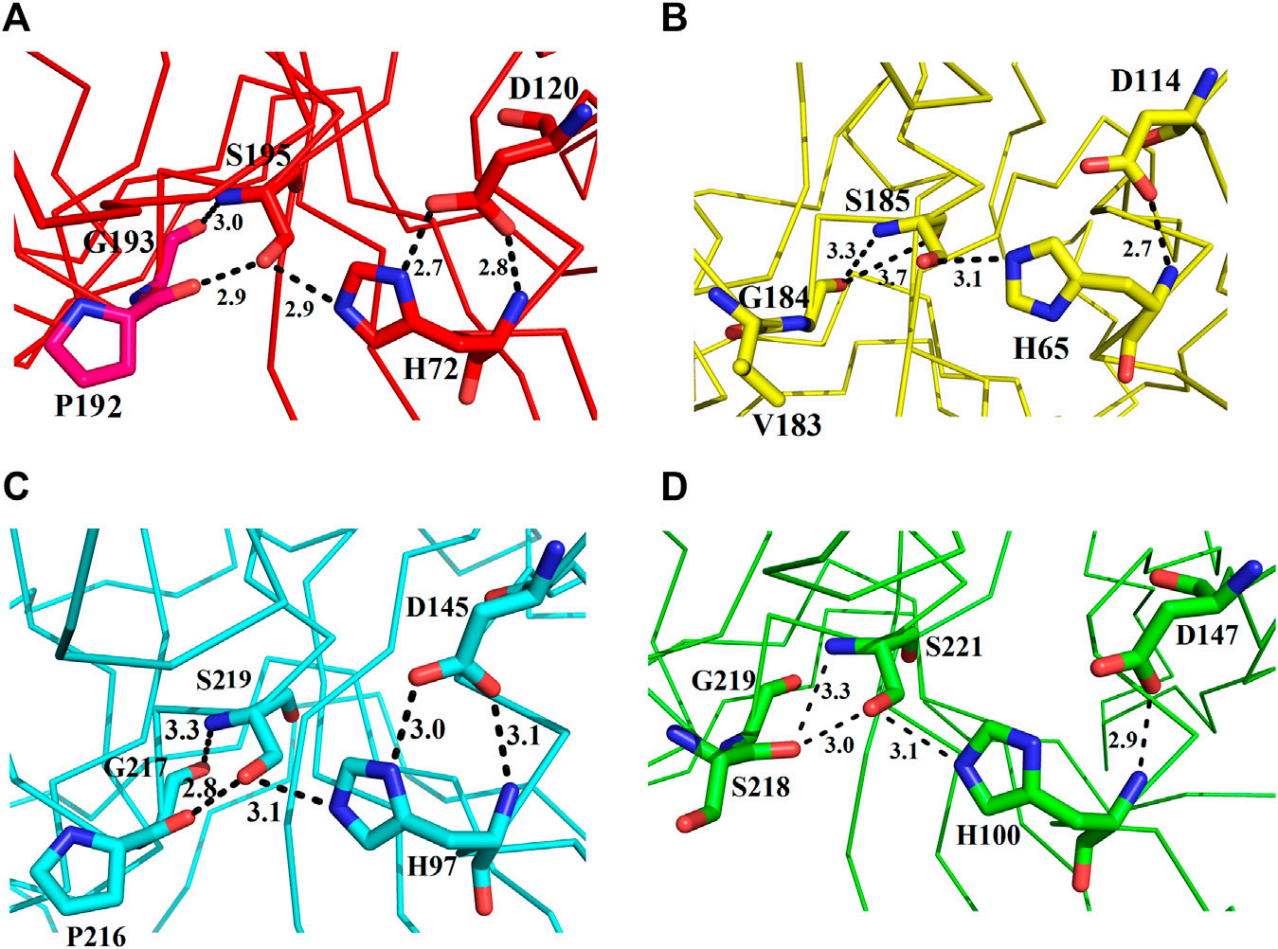
Representation of amino acid residues that make the ETs inactive: **(A)** ETA, **(B)** ETB, **(C)** ETE, and **(D)** ETD_model.

These enzymes are usually inactive in their free state due to the hydrogen bonds between Pro192 and G193 (donor–acceptor distances of 2.9–3.0 Å, ETA), V183 and G184 (donor–acceptor distances of 3.3–3.7 Å, ETB), P216 and G217 (donor–acceptor distances of 2.8–3.3 Å, ETE), and S218 and G219 (donor–acceptor distances of 3.0–3.3 Å, ETD (Figures 7A–D). A mean distance of 3.0 Å (donor–acceptor) is required for hydrogen bond formation in protein secondary structure (Rajagopal et al., 2005; Dev et al., 2015). Depending on the distance between donor and acceptor, the hydrogen bond can be classified into three main classes (Jeffrey, 1997). These are strong hydrogen bonds (often covalent) (donor–acceptor distances of 2.2–2.5 Å), medium hydrogen bonds (largely electrostatic) (2.5–3.2 Å), and weak hydrogen bond (electrostatic) (3.2–4.0 Å). Keeping this in view, the hydrogen bond between active site serine residue and other amino acid residues (Pro, Gly, Val, and Ser) are classified as medium (ETA and ETE) and weak (ETB and ETD). Thus ETA and ETE are inactive in their native states and ETB and ETD are active. According to Steitz and Shulman (1982), most of the serine proteinases are inactive in their native state and become active only when substrate binds to these enzymes.

### Mechanism of Action

**FIGURE 8:**
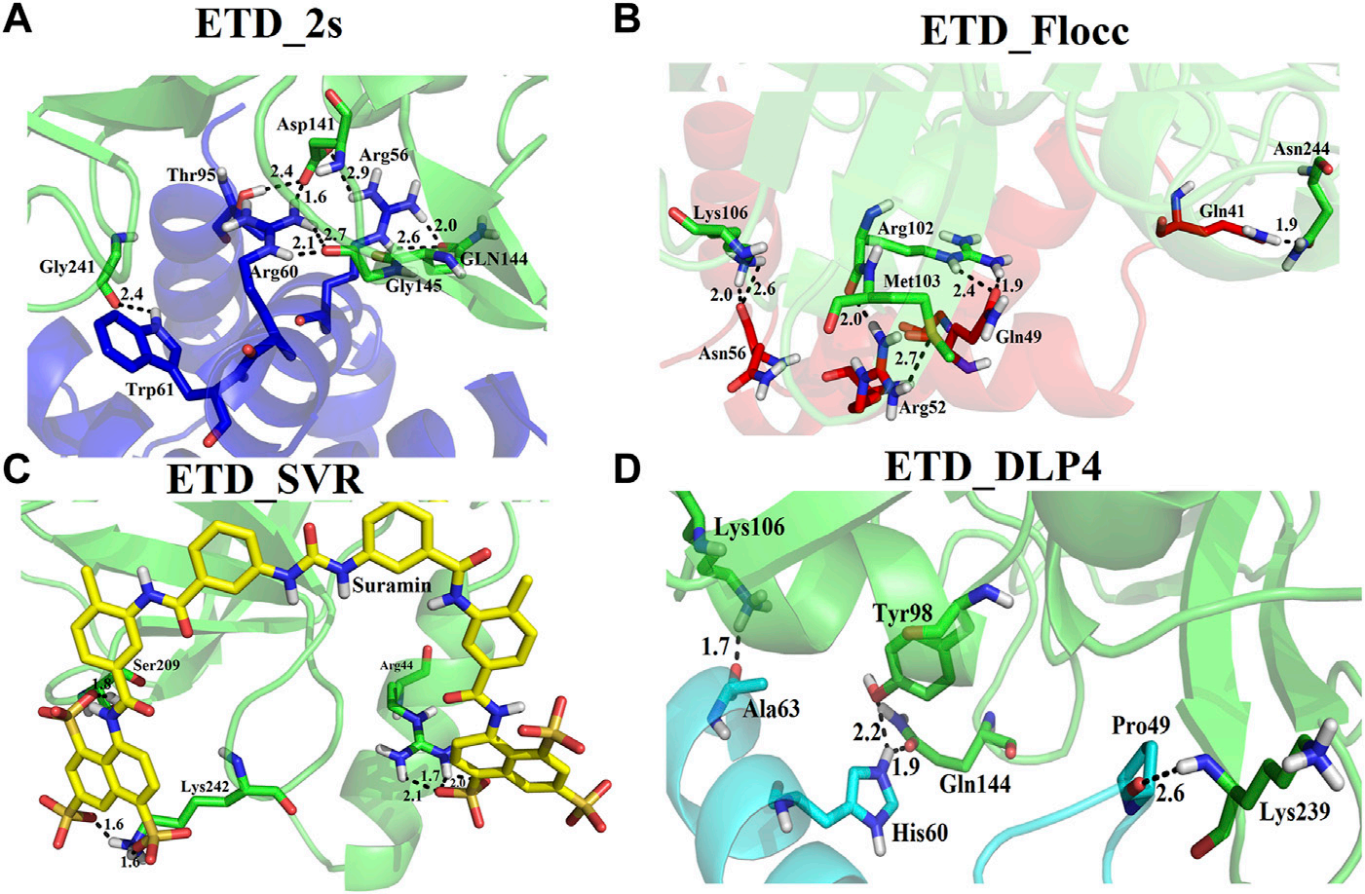
Proposed mechanisms for ETs. **(A)** The catalytic triad (His72, Asp120, and Ser195) and the amino acid residues Pro192, Gly193 (red sticks), and hydrolytic water (green sphere); the enzyme is inactive in this state. **(B)** The enzyme (red) becomes active after binding to the substrate (green), by disrupting the bond with Pro192. **(C)** The enzyme attacks the substrate (peptide) (green sticks) carboxyl group. **(C)** Tetrahedral intermediate. **(D)** Donation of H+ by His72 to peptide for leaving group, and breakage of the tetrahedral intermediate and release of the first product. **(E)** Splitting of the hydrolytic water molecule into H+ and OH−. **(F)** The OH− attacks the bond between Ser195 and the peptide C-terminal end and liberates this part from the enzyme. **(G)** ET (red) and enzyme (green) complex.

The ETs use the same catalytic mechanisms as used by the other serine proteinases-like chymotrypsin to hydrolyze their substrate (Hartley and Kilby, 1954; Polgár, 1971; Katona et al., 2002). However, there is no oxyanion hole formation in ETs (Papageorgiou et al., 2000). The three catalytic amino acid residues (H57, D102, and S195—catalytic triad) are fully conserved in all ETs (Figure 1) and also in other serine proteinases (Birktoft et al., 1976; Steitz and Shulman, 1982; Bagley and Altman, 1996). These ETs follow the general acid–base catalysis (Anderson et al., 1961; Polgár and Bender, 1969; Polgár, 1971). They are inactive in their free state due to the hydrogen bond formed between active site serine and some other specific residues (Figures 7A-D, Figure 8). A twist of 180° in these amino acid residues (Figure 8B) results when the N-terminal helix binds to a specific epidermal receptor, that make the toxins active (Vath et al., 1999 and 1997). His72 then takes a proton from Ser195 (Figure 8B) and makes the serine residue a strong electrophile (Figure 10C) that attacks on the sessile peptide bond between Glu381 and G382 (Figure 8C). This leads to the construction of a tetrahedral intermediate which is formed between Ser195 and desmoglein-1 (substrate of ETs) (Figure 8D). This intermediate is unstable, and electrons rearrangement leads to breakage: the C-terminus of the substrate is detached and released (Figure 8E). The N-terminus of the substrate is still attached to the enzyme (Figure 8F). An attack from OH− of water on the bond between the enzyme and N-terminus of the substrate causes breakage of the N-terminus of the substrate, and this part also departs from the enzyme and is restored to its original state.

### Inhibition Study of Exfoliative Toxin D by Natural and Synthetic Inhibitors

**FIGURE 9:**
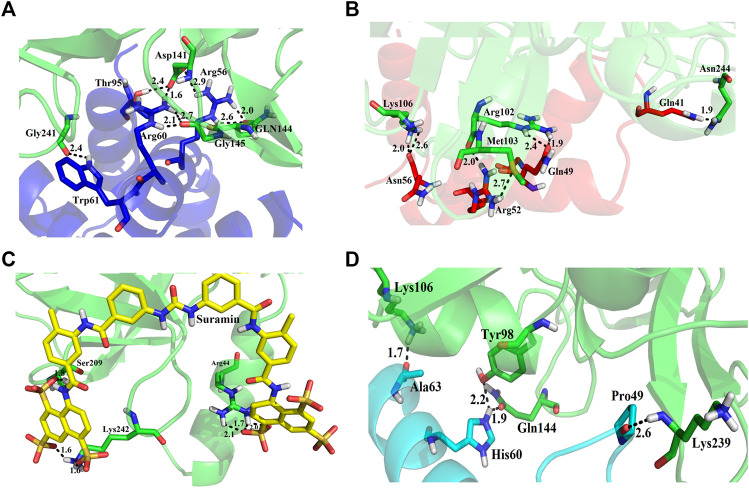
Inhibition of ETD_*Sa* (green sticks) by **(A)** 2S albumin (blue sticks), **(B)** flocculating protein (red sticks), **(C)** suramin (yellow sticks), and **(D)** DLP-4 (cyan sticks).

**FIGURE 10:**
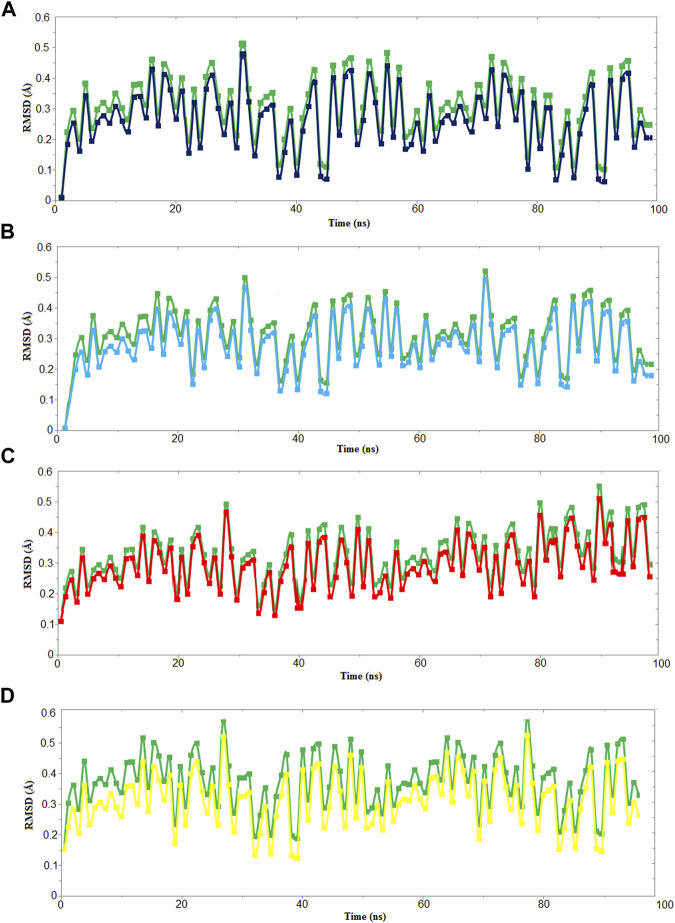
RMSD vs time graph for 100 ns MD simulation of ETD_*Sa* (green) with **(A)** 2S albumin, **(B)** flocculating protein (black), **(C)** suramin (red), and **(D)** DLP4 (yellow).

**FIGURE 11:**
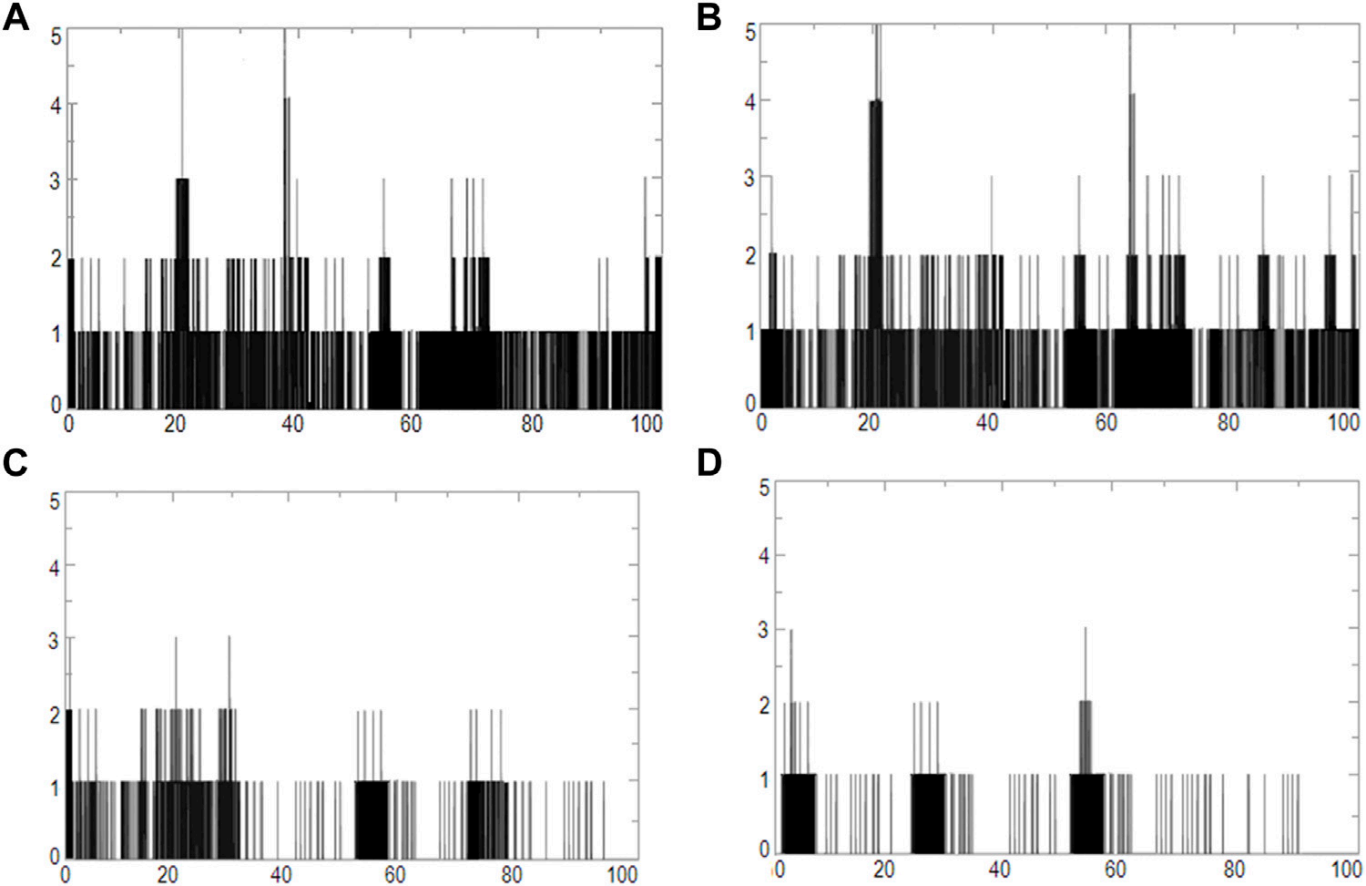
Graphs of hydrogen bonds plotted vs time for 100 ns range simulations of ETD_Sa and **(A)** 2S albumin, **(B)** flocculating protein, **(C)** suramin, and **(D)** DLP4.

We used four inhibitors: two natural [2S albumin (PDB ID: 5DOM, Ullah et al., 2015) and flocculating protein [PDB ID: 5WUZ, Kini et al., 2016 (Unpublished) *Moringa oleifera*] and two synthetics [suramin (PubChem CID: 5361) and defensin-like peptide 4 (DLP4) (UniProtKB ID: W5U4X3)]. The 2S albumin and flocculating protein are highly positively charged with several arginine residues located on their surfaces. Both of these inhibitors bind to the middle and C-terminal regions of the ETD (Figures 9A,B). The 2S albumin and flocculating protein make six hydrogen bonds (Supplementary Figures S13A–C). However, 2S albumin makes two salt bridges and 64 nonbonded contacts, while the flocculating protein makes one salt bridge and 84 nonbonded contacts (Supplementary Figures S13A–C). In the case of synthetic inhibitors, suramin binds to the N- and C-terminal of ETD and makes five hydrogen bonds and 47 nonbonded contacts (Figure 9C; Table 4), while DLP4 binds to the middle and C-terminal regions of ETD and make four hydrogen and 40 nonbonded contacts (Figure 9D; Supplementary Figures S14A–C). The 2S albumin and flocculating protein have already been used as inhibitors for Coronavirus 3CL^Mpro^ (Ullah and Ullah, 2021), while suramin has been shown to inhibit the function of human thrombin, snake venom thrombin like enzymes, and phospholipases A2 enzymes (Monteiro et al., 2004; Murakami et al., 2005; Fernandes et al., 2007; Lima et al., 2009; Ullah et al., 2018). The inhibition of ETD from *S. hyicus* has been done with DLP4 (Li et al., 2020; Ma et al., 2021). The results of MD simulation (GROMACS) indicate that the interactions between ETD_*Sa* and all the four ligands were stable throughout the simulation process (Figure 10), and 2S albumin and flocculating proteins made 4–6 bonds while suramin and DLP4 formed 2–4 hydrogen bonds (Figure 11).

**TABLE 4:** List of protein (ETD)–ligand (suramin) interactions.

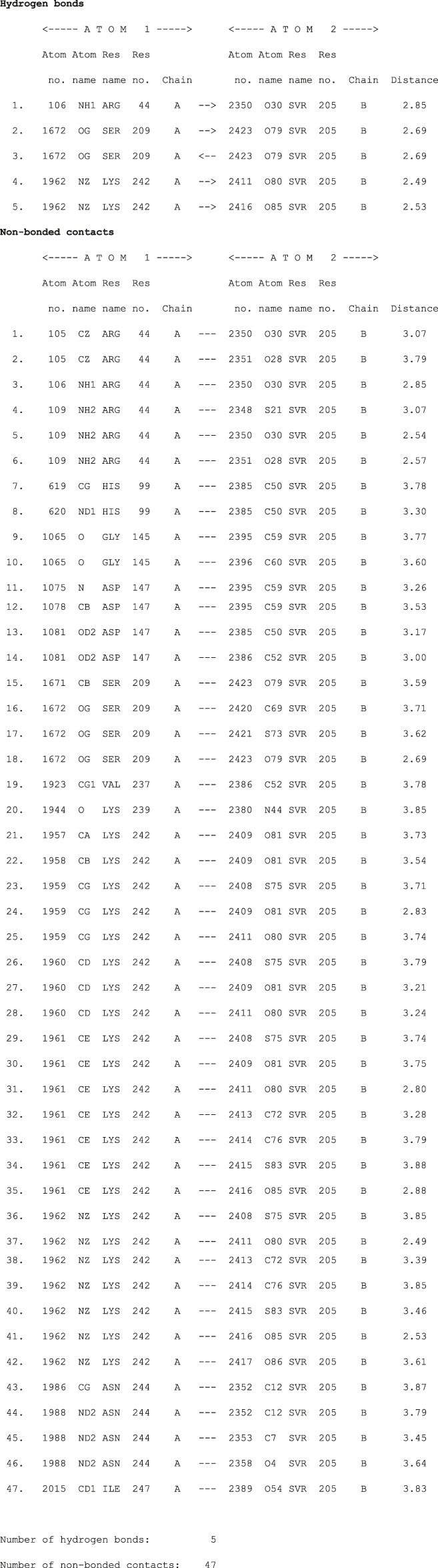

### Differences Between Exfoliative Toxins and Other Serine Proteinases

The ETs differ from other serine proteinases by possessing an N-terminal α-helix that is highly charged (Vath et al., 1997; Cavarelli et al., 1997), and they also have two amino acid residues [proline (192)–glycine (193), ETA and valine (183)–glycine (184), ETB] which make a hydrogen bond with the active site serine and make them inactive (Ladhani, 2003). In the catalytic reactions of ETs, there is no formation of oxyanion hole formation (Papageorgiou et al., 2000). The serine proteinases from other organisms are highly stable by having many disulfide bridges (Ullah et al., 2018); however, the ETs lack any such disulfide bridges (Mariutti et al., 2015; Piemont et al., 1986; Papageorgiou et al., 2000). The serine proteinases are highly glycosylated, while ETs do not have any carbohydrates in their 3D structure (Piemont et al., 1986; Papageorgiou et al., 2000; Mariutti et al., 2015).

*S. aureus* not only causes diseases in human beings but also in other organisms like small piglets (Schwarz et al., 2021; Malik et al., 2021) and dogs (Sahin-Tóth et al., 2021). These bacteria show resistance to methicillin and are also called methicillin-resistant *S. aureus* (Hirose et al., 2019). Their resistance to methicillin is a serious issue because these bacteria are the main cause of skin infections in animals (Hirose et al., 2019; Del Giudice, 2020). To find a new treatment for skin diseases caused by *S. aureus*, many researchers around the world are trying to find out novel inhibitors for the toxins produced by these bacteria. Mohan and Venugopal (2020) have carried out molecular binding and simulation studies of flavonoids those inhibit the ETA of *S. aureus* and have come to know that these make three hydrogen bonds with the ETA. Li et al., 2020 have used DLP4 and its derivatives as antimicrobial agents against *S. aureus* (Li et al., 2020)*.* Suramin is used as an antitrypanosomal drug, and it has been shown to inhibit human thrombin (Lima et al., 2009), snake venom phospholipases A2 (Salvador et al., 2018), snake venom serine proteinases (Ullah et al., 2018), nucleocapsid protein from novel bunyavirus (Jiao et al., 2013), RNA-dependent RNA polymerase of murine norovirus (Mastrangelo et al., 2012), and pyruvate kinase of *Leishmania mexicana* (Morgan et al., 2011). In the majority of the studies carried out using suramin as inhibitor, it has been found that it binds to the C-terminal part of the protein (Lima et al., 2009; Ullah et al., 2018). In the current study, suramin was found to bind to the C-terminal of ETD also (Figure 11C). The 2S albumin seed storage proteins (Ullah et al., 2015) and flocculating protein (Sousa et al., 2020) from *M. oleifera* are highly positively charged proteins. In our previous study, we had shown that these proteins bind to SARS-CoV-2 3CL M ^pro^ in-between domain II and III and restrict their moment (Ullah and Ullah, 2021). This may result in the inactivation of this enzyme. In the present study, the 2S albumin seed storage proteins and flocculating protein bind in-between the N- and C-termini of ETD (Figures 11A,B) and may restrict the moments in these parts.

## Conclusions and Future Perspectives

In conclusion, the model-based structure characterization of ETD_*Sa* and its sequence and structural comparison with other ETs were studied in this work. ETD_*Sa* displays a high sequence identity with the other ETs. The build ETD_*Sa* model also aligned well to the 3D structures of other ETs, with some differences that were confined to the loop regions. The differences in amino acid residues in the loop regions cause a different surface charge distribution to these enzymes which may also convey variable substrate specificity to these enzymes. The proposed mechanism of the ET was elucidated based on their 3D structure. The inhibition study of these enzymes by natural and synthetic inhibitors will further facilitate the treatment of SSSS. Finally, for further validation of the current study, the 3D structure of ETD_*Sa* may be determined by X-ray crystallography.

## Data Availability

The datasets presented in this study can be found in online repositories. The names of the repository/repositories and accession number(s) can be found in the article/Supplementary Material.
