# Supplementary material for: Three-Dimensional Structure Characterization and Inhibition Study of Exfoliative Toxin D From Staphylococcus aureus
Source: Front Pharmacol. 2022 Feb 18;13:800970. doi: 10.3389/fphar.2022.800970 (PMC8895341; doi:10.3389/fphar.2022.800970)
Supplement: Supplementary file 1 [file DataSheet1.DOCX]

**Supplementary materials**

| **Protein (Template)** | **Identity (%)** | **GMQE** | **QMEANDisCO Global** |
| --- | --- | --- | --- |
| 5C2Z | 63.71 | 0.79 | 0.83 + 0.05 |
| 6E0U | 69.01 | 0.77 | 0.81 + 0.05 |
| 1QTF | 64.17 | 0.77 | 0.82 + 0.05 |
| 1DUE | 50.79 | 0.60 | 0.60 + 0.06 |

**Table S1:** Comparative modeling statistics

**Table S2:** RMSD values of all generated models

| **Model produced using the template with PDB ID** | **RMSD value (Å)** |
| --- | --- |
| 1DUE align to 6E0U | 0.722 |
| 1DUE align to 1QTF | 0.517 |
| 1DUE align to 5C2Z | 0.562 |
| 6E0U align to 1QTF | 0.552 |
| 6E0U align to 5C2Z | 0.481 |
| 1QTF align to 5C2Z | 0.496 |

**
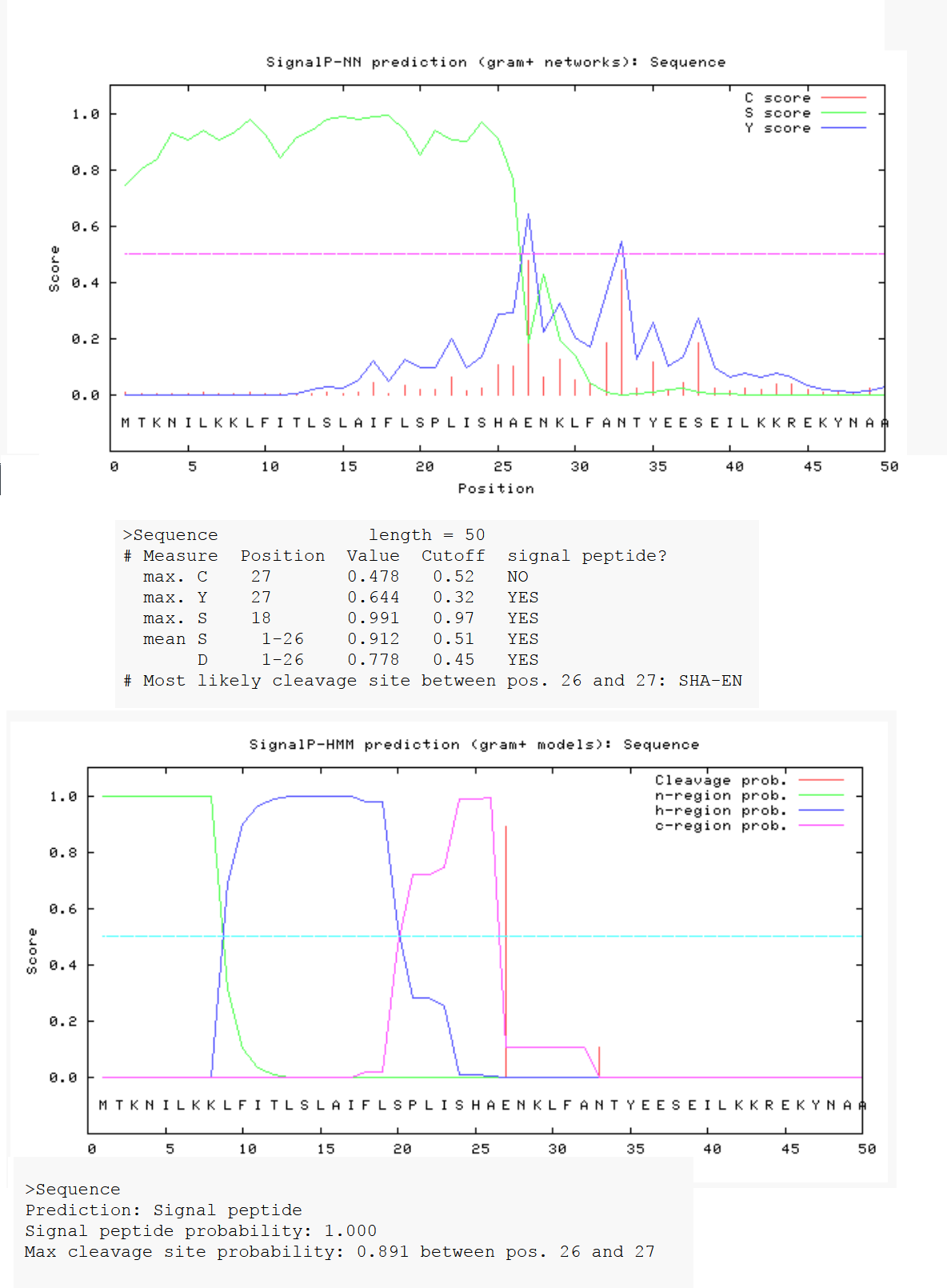
**

**Figure S1:** Results of prediction of signal peptide using the SignalP 3.0

**
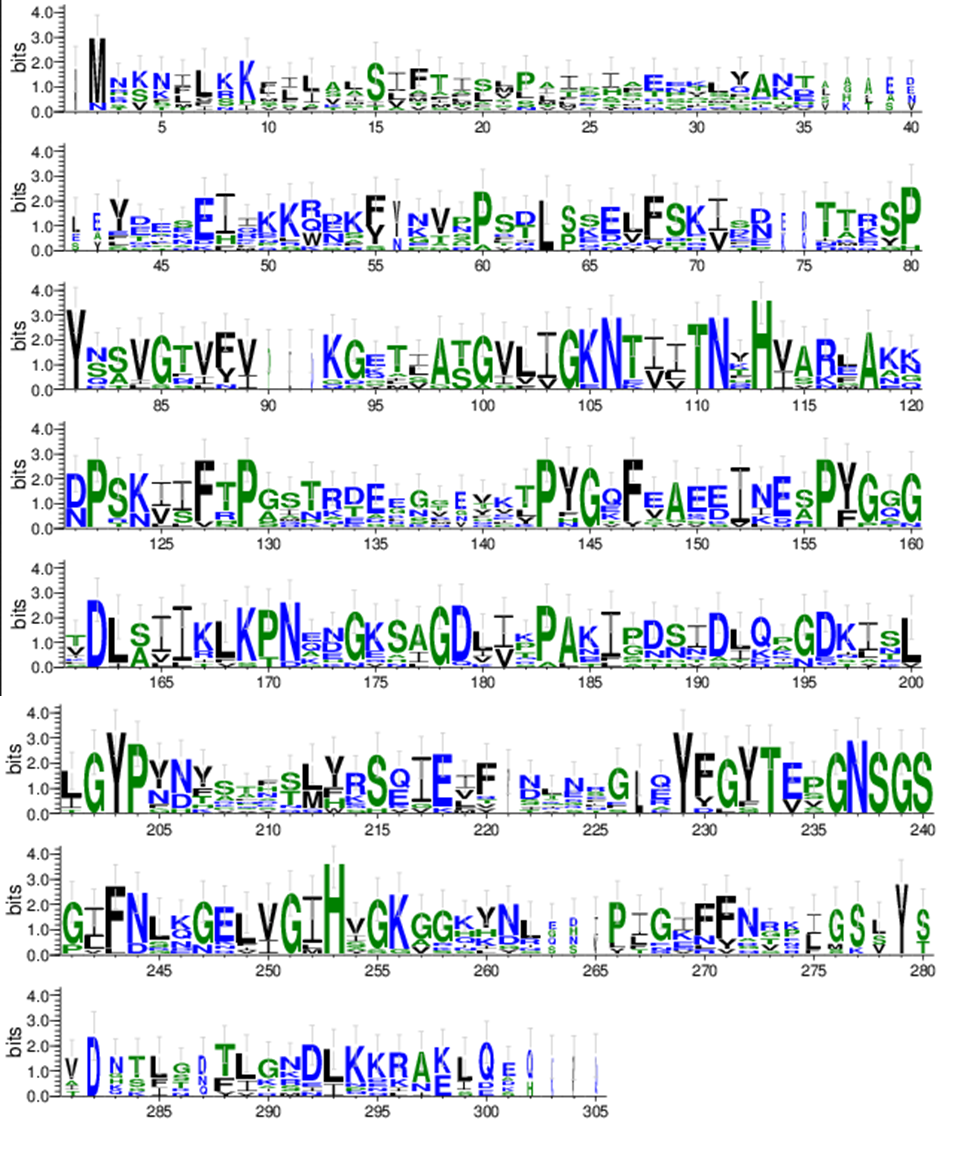
**

**Figure S2:** Sequence logo generated from the aligned sequence


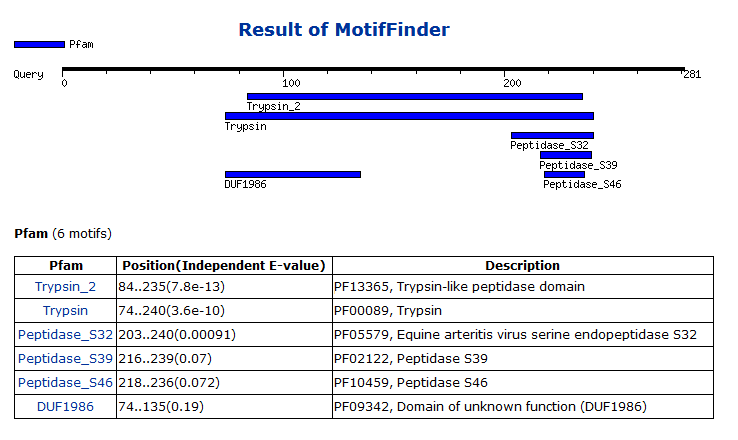


**Figure S3:** Results from motif finder for ETD_*Sa*.


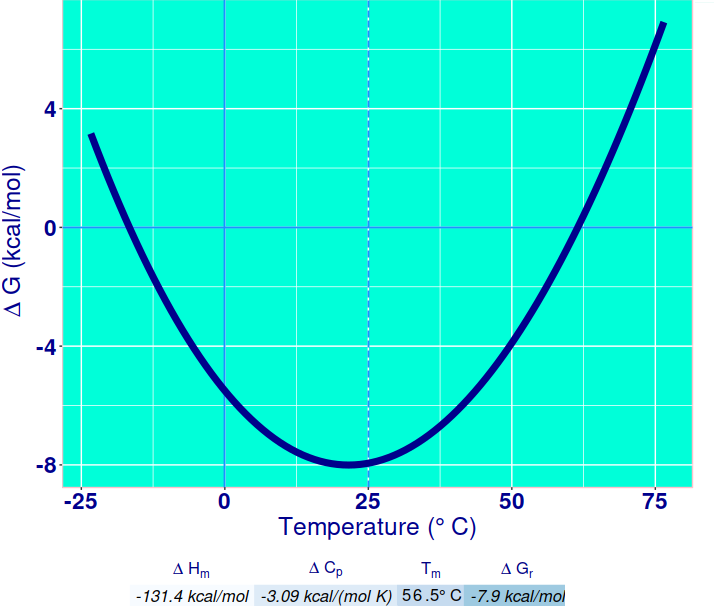


**Figure S4:** Thermogram (melting temperature) for ETD_*Sa*.


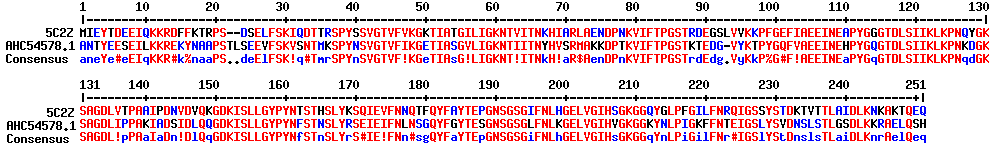


**Figure S5:** Sequence alignment between the template (PD ID: 5C2Z) and the target protein (ETD_*Sa*: Accession No. AHC54578.1), Red- High consensus color, Blue-Low consensus color, Black-Neutral color


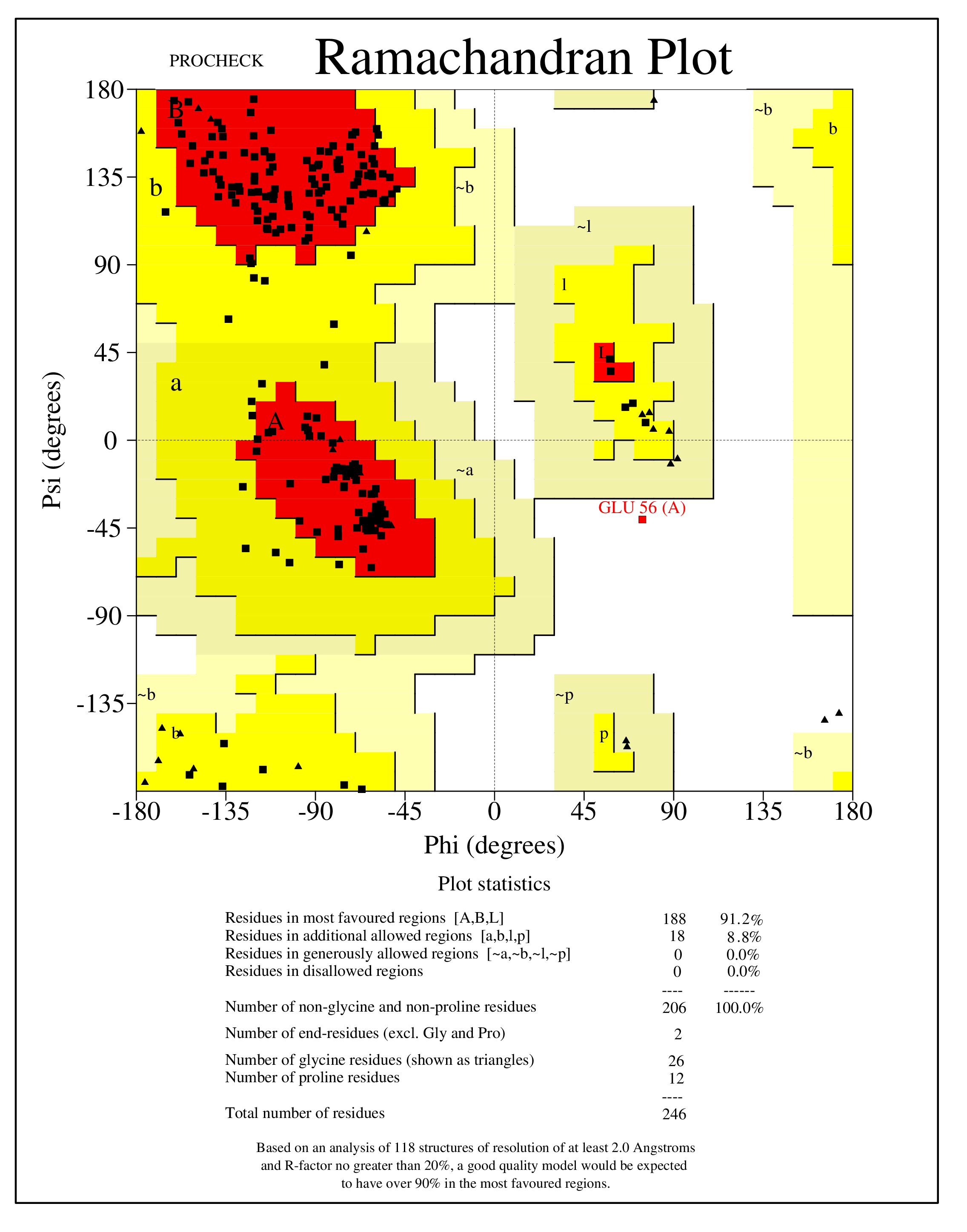


**Figure S6:** Ramchandran plot for the build ETD_*Sa* model.


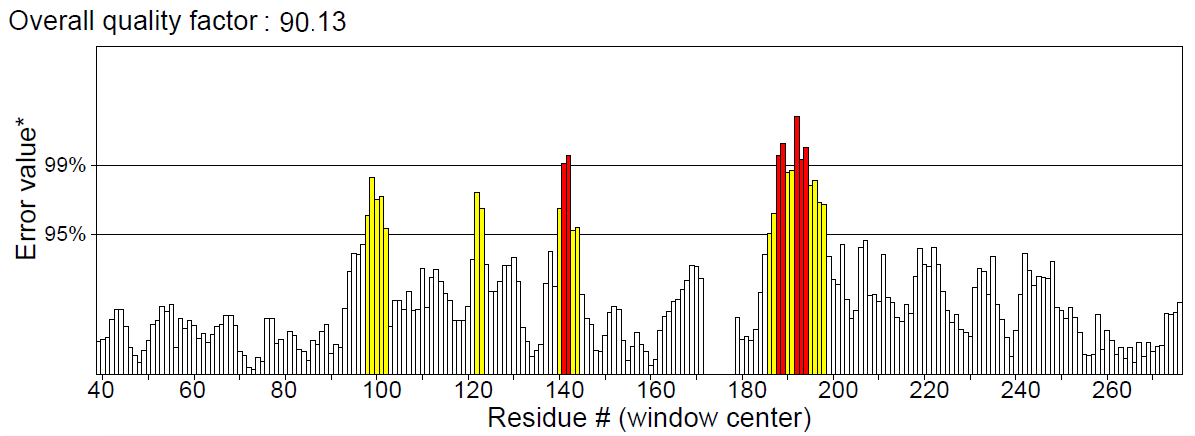


**Figure S7:** ERRAT plot for the build ETD_*Sa* model.


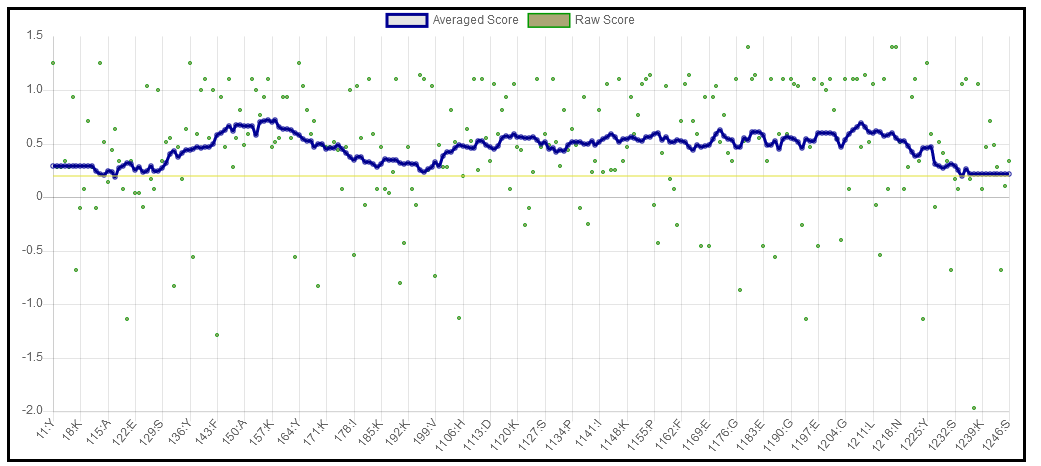


**Figure S8:** Verify3D plot for the build ETD_*Sa* model.


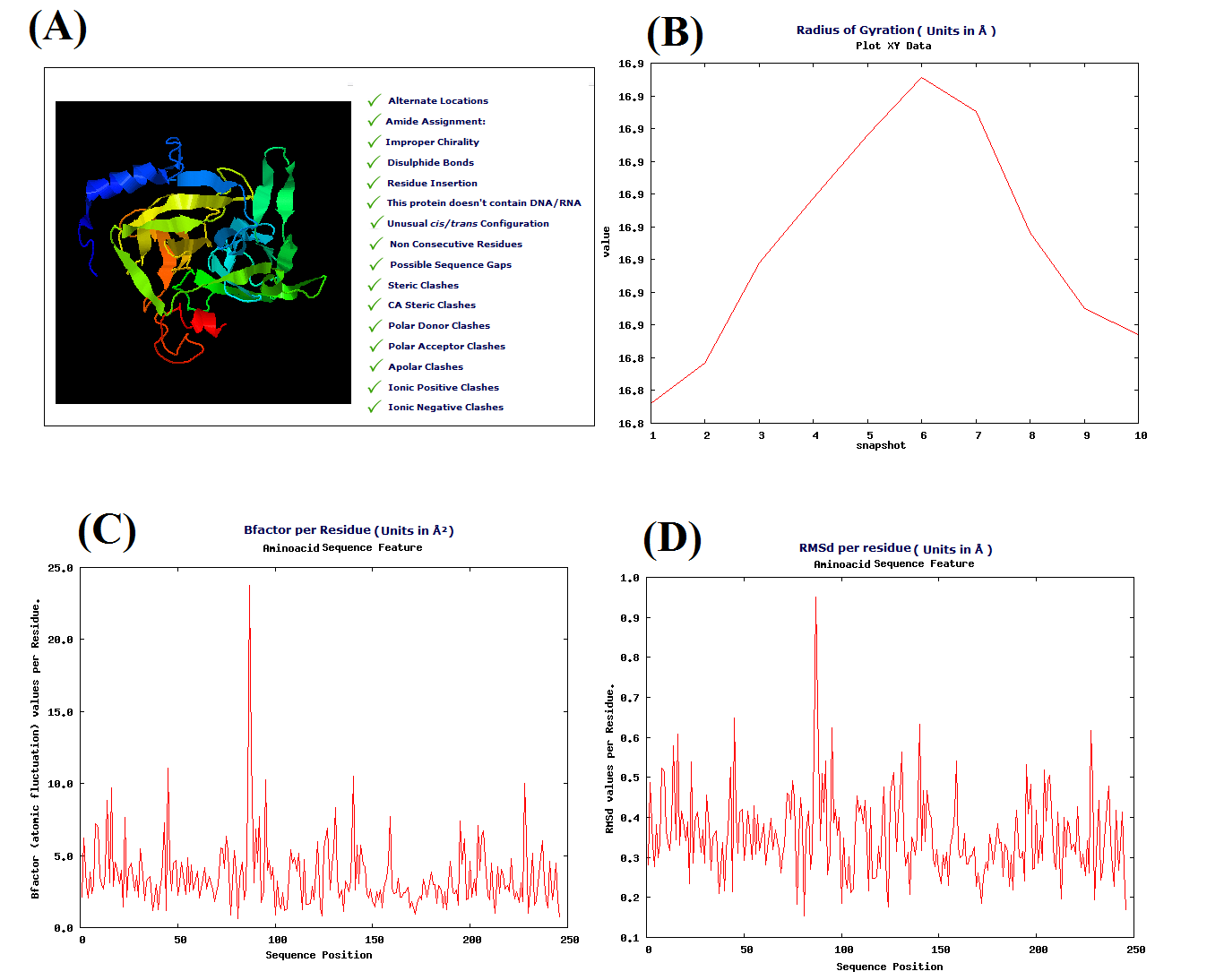


**Figure S9. Molecular dynamic simulation analysis of ETD_*Sa.*** **(A)** Structural parameters; **(B)** Radius of

Gyration; **(C)** B-Factor per residue; **(D)** RMSD per residue.


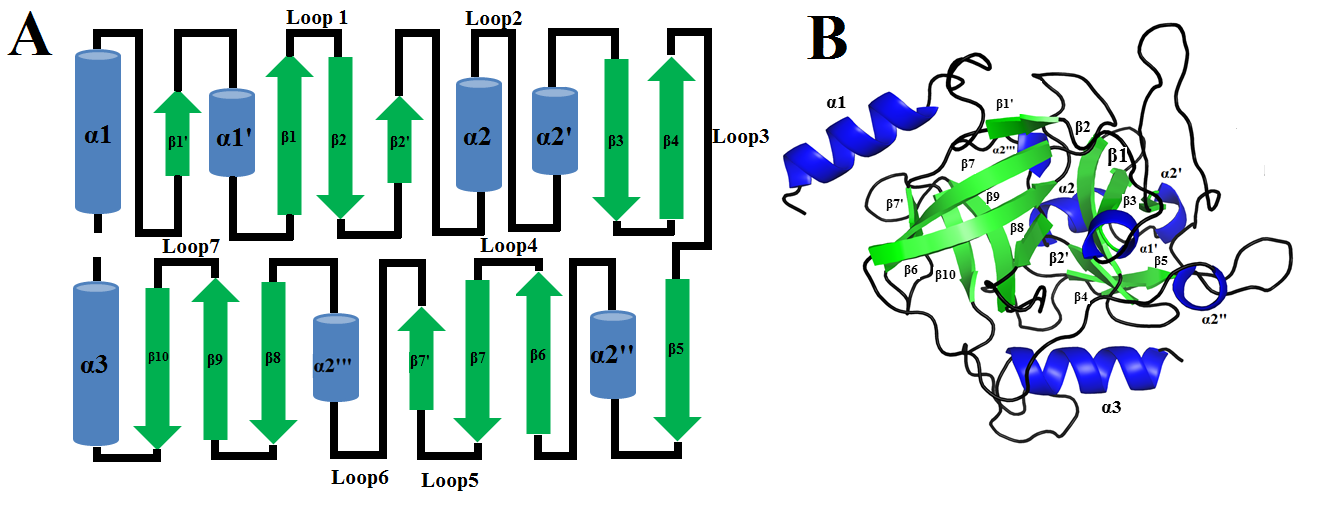
**Figure S10:** **Topology diagram of ETD_*Sa*.** The beta strands (named β1–β10) was represented as arrows and alpha helices (named α1–α3) as cylinders. The short beta strands and alpha helices are shown with primes. The secondary structures and the amino acid residues in alpha helices and beta strands were assigned using the program DSSP from primary sequence and were confirmed by PyMOL from the tertiary structure **(B)** Cartoon representation of ETD_*Sa.*


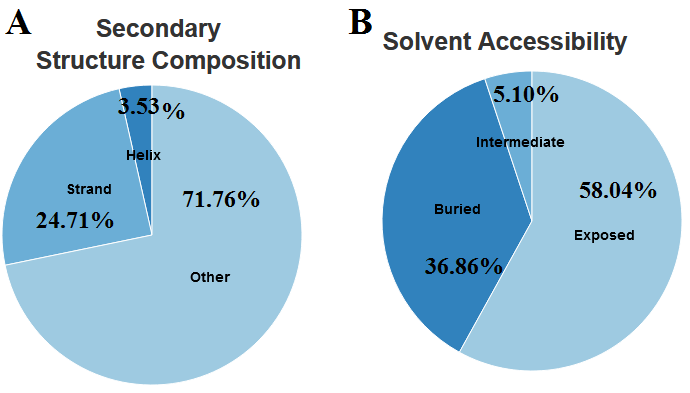


**Figure S11:** **(A)** Secondary structure composition and **(B)** solvent accessibility in ETD_*Sa* structure.


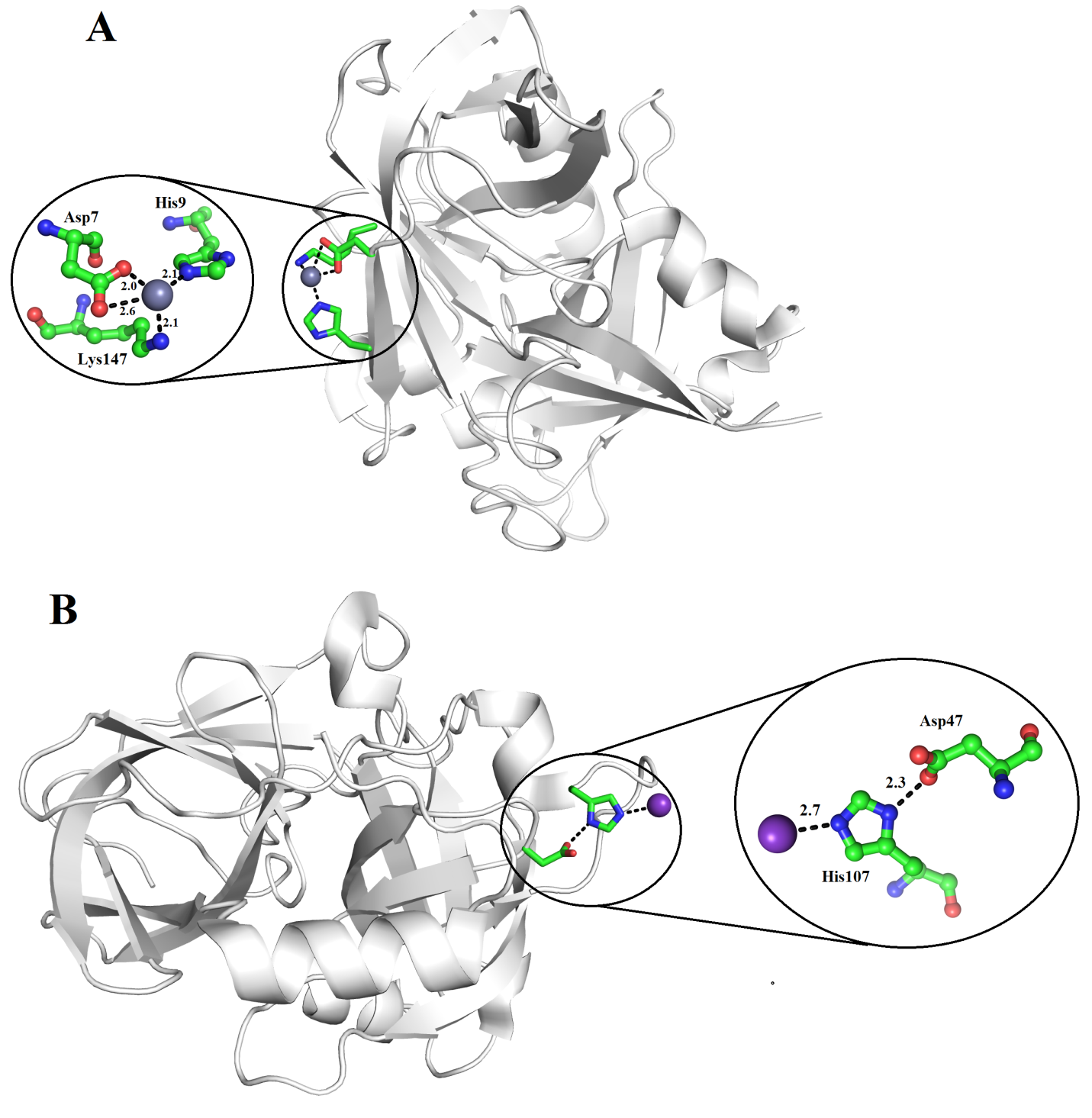
 **Figure S12**: Metal ions binding in ETs (A) Zinc ion in the Crystal structure of an alkaline form of v8 protease from Staphylococcus aureus (B) Potassium ion in the structure of V8 Protease from Staphylococcus aureus, PDB ID: 1QY6.


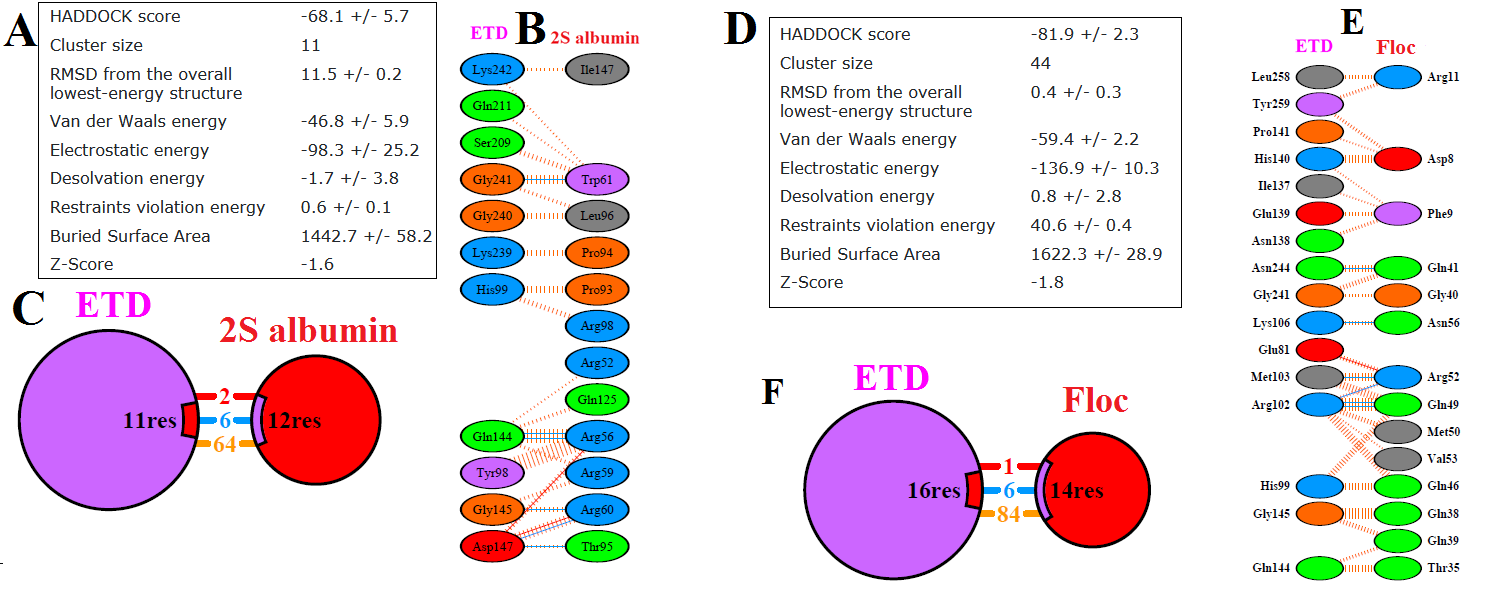


**Figure S13:** **Interaction of ETD_*Sa* with 2S albumin and flocculating proteins** **(A & D)** HADDOCK molecular docking statistics **(B &E)** Interactions of amino acid residues from ETD_Sa and 2S albumin and flocculating proteins **(C &F)** Numbers of hydrogen bonds, salt bridges and non-bonded contacts.


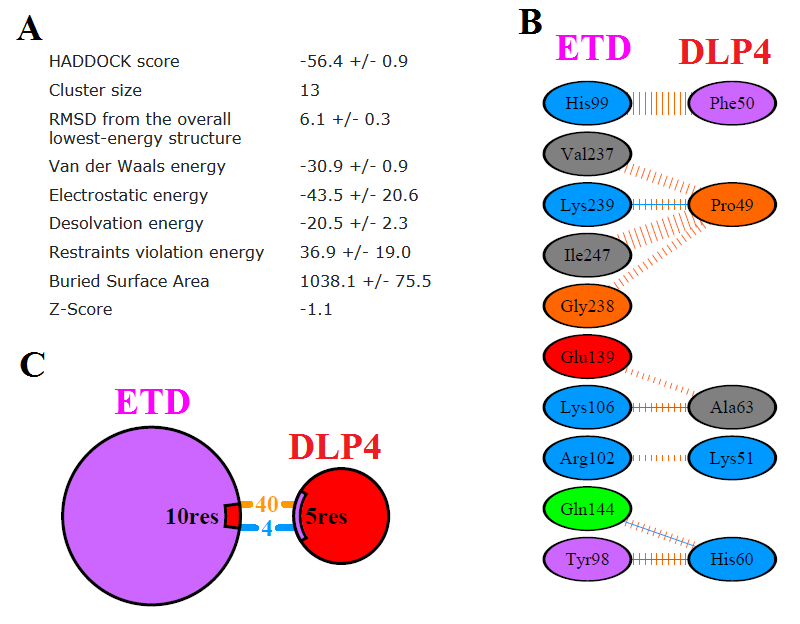


**Figure S14:** **Interaction of ETD_*Sa* with DLP4** **(A)** HADDOCK molecular docking statistics **(B )** Interactions of amino acid residues from ETD_Sa and DLP4 **(C &F)** Numbers of hydrogen bonds, salt bridges and non-bonded contacts.
